# The *Borrelia burgdorferi* Adenylate Cyclase, CyaB, Is Important for Virulence Factor Production and Mammalian Infection

**DOI:** 10.3389/fmicb.2021.676192

**Published:** 2021-05-25

**Authors:** Vanessa M. Ante, Lauren C. Farris, Elizabeth P. Saputra, Allie J. Hall, Nathaniel S. O’Bier, Adela S. Oliva Chávez, Richard T. Marconi, Meghan C. Lybecker, Jenny A. Hyde

**Affiliations:** ^1^Department of Microbial Pathogenesis and Immunology, Texas A&M University Health Science Center, Bryan, TX, United States; ^2^Department of Biology, University of Colorado at Colorado Springs, Colorado Springs, CO, United States; ^3^Department of Microbiology and Immunology, Virginia Commonwealth University Medical Center, Richmond, VA, United States; ^4^Department of Entomology, Texas A&M University, College Station, TX, United States

**Keywords:** adenylate cyclase (AC), CyaB, *Borrelia burgdorferi* spirochete, Lyme disease, secondary messenger, small RNA (sRNA), cyclic nucleotides

## Abstract

*Borrelia burgdorferi*, the causative agent of Lyme disease, traverses through vastly distinct environments between the tick vector and the multiple phases of the mammalian infection that requires genetic adaptation for the progression of pathogenesis. Borrelial gene expression is highly responsive to changes in specific environmental signals that initiate the RpoS regulon for mammalian adaptation, but the mechanism(s) for direct detection of environmental cues has yet to be identified. Secondary messenger cyclic adenosine monophosphate (cAMP) produced by adenylate cyclase is responsive to environmental signals, such as carbon source and pH, in many bacterial pathogens to promote virulence by altering gene regulation. *B. burgdorferi* encodes a single non-toxin class IV adenylate cyclase (*bb0723*, *cyaB*). This study investigates *cyaB* expression along with its influence on borrelial virulence regulation and mammalian infectivity. Expression of *cyaB* was specifically induced with co-incubation of mammalian host cells that was not observed with cultivated tick cells suggesting that *cyaB* expression is influenced by cellular factor(s) unique to mammalian cell lines. The 3′ end of *cyaB* also encodes a small RNA, SR0623, in the same orientation that overlaps with *bb0722*. The differential processing of *cyaB* and SR0623 transcripts may alter the ability to influence function in the form of virulence determinant regulation and infectivity. Two independent *cyaB* deletion B31 strains were generated in 5A4-NP1 and ML23 backgrounds and complemented with the *cyaB* ORF alone that truncates SR0623, *cyaB* with intact SR0623, or *cyaB* with a mutagenized full-length SR0623 to evaluate the influence on transcriptional and posttranscriptional regulation of borrelial virulence factors and infectivity. In the absence of *cyaB*, the expression and production of *ospC* was significantly reduced, while the protein levels for BosR and DbpA were substantially lower than parental strains. Infectivity studies with both independent *cyaB* mutants demonstrated an attenuated phenotype with reduced colonization of tissues during early disseminated infection. This work suggests that *B. burgdorferi* utilizes *cyaB* and potentially cAMP as a regulatory pathway to modulate borrelial gene expression and protein production to promote borrelial virulence and dissemination in the mammalian host.

## Introduction

*Borrelia burgdorferi*, the causative agent of Lyme disease, is an emerging infectious disease that causes a robust inflammatory multistage disease and accounts for over 80% of all vector-borne illnesses in the United States (Radolf et al., 2012; Steere et al., 2016; Rosenberg et al., 2018; Stanek and Strle, 2018). Localized disease presents as flu-like symptoms and is frequently associated with an erythema migrans “bull’s-eye” rash (Steere et al., 2016; Stanek and Strle, 2018). If left untreated, the pathogen disseminates to specific tissues with systemic symptoms developing including arthritis, carditis, and encephalomyelitis (Hu, 2016; Steere et al., 2016; Stanek and Strle, 2018). Patients experience severe morbidity due to ongoing fatigue and malaise as a result of the inflammatory response elicited by *B. burgdorferi*. To date, no human vaccine is available and therapeutics for late stage disease are limited.

*Borrelia burgdorferi* lacks classically defined virulence factors, such as secretion systems and toxins, and instead relies on dynamic genetic regulation and antigenic variability to invade multiple tissue types and evade the immune system (Radolf et al., 2012; D. Scott Samuels and Samuels, 2016). Many studies have noted the responsiveness of *B. burgdorferi* to environmental signals, such as temperature, pH, O_2_, CO_2_, and osmotic stress, as it travels from the tick vector to the mammalian host, but mechanisms of direct environmental detection remain unknown (Stevenson et al., 1995; Carroll et al., 1999, 2000; Konkel and Tilly, 2000; Yang et al., 2000; Tokarz et al., 2004; Seshu et al., 2004a; Hyde et al., 2007; Bontemps-Gallo et al., 2016; Popitsch et al., 2017). The BosR–Rrp2–RpoN–RpoS signaling cascade responds to changing environmental cues to allow borrelial adaptation during early mammalian infection and resistance to innate immunity by altering the outer membrane lipoprotein composition (Caimano et al., 2007, 2004; Smith et al., 2007; Ouyang et al., 2008; Hyde et al., 2009, 2010; Blevins et al., 2009; Ouyang et al., 2009, 2011; Caimano et al., 2019). Transcription of *rpoS* is regulated by a transcription complex composed of the RNA polymerase, the sigma factor RpoN, the phosphorylated Response regulator protein (Rrp2), and the Borrelia oxidative stress regulator (BosR) (Yang et al., 2003; Smith et al., 2007; Blevins et al., 2009; Hyde et al., 2009; Ouyang et al., 2009, 2011; Hyde et al., 2010). The borrelial RpoS regulon includes outer surface lipoproteins DbpA, OspC, and BBK32 and other factors important for tick to mouse transmission and survival in mammalian hosts (Hübner et al., 2001; Caimano et al., 2005, 2007; Yang et al., 2005; He et al., 2007, 32).

Secondary messengers are a mechanism used by bacterial pathogens, such as *B. burgdorferi*, to modulate gene expression and posttranscriptional regulation in response to environmental signals by altering the function of bound proteins (Rogers et al., 2009; McDonough and Rodriguez, 2011; Ye et al., 2014; Savage et al., 2015; Aline Dias da et al., 2020; Yin et al., 2020). In *B. burgdorferi*, the second messenger cyclic di-adenosine monophosphate (c-di-AMP) is essential for *in vitro* growth and the production of mammalian virulence factors (Ye et al., 2014; Savage et al., 2015). Cyclic di-guanosine monophosphate (c-di-GMP) is a key component of the Hk1–Rrp1 two-component system pathway involved in mammal to tick transmission, midgut survival, motility, and glycerol utilization by *B. burgdorferi* (He et al., 2011; Sultan et al., 2011; Novak et al., 2014; Caimano et al., 2015; Bontemps-Gallo et al., 2016; Zhang et al., 2018). c-di-GMP is produced by Rrp1 and bound by PlzA to positively regulate glucose metabolism (Rogers et al., 2009; Freedman et al., 2010; Sultan et al., 2010; Kostick et al., 2011; He et al., 2014; Mallory et al., 2016; Kostick-Dunn et al., 2018; Zhang et al., 2018). Another second messenger cyclic adenosine monophosphate (cAMP) has received less attention in *B. burgdorferi*, but has been found to support virulence in other pathogenic bacteria (McDonough and Rodriguez, 2011). cAMP is generated by adenylate cyclases (ACs) to modulate regulation of the bacteria or the host cell depending on the six classes of AC. cAMP can bind to cAMP receptor proteins (CRP) often resulting in a conformation change that promotes efficient binding of specific DNA sites and transcription of numerous genes. *B. burgdorferi* encodes a single class IV AC (*bb0723*), annotated as *cyaB*, which is the smallest of the classes, is highly thermostable, and has been identified in only three other bacterial species (Sismeiro et al., 1998; Casjens et al., 2000; Gallagher et al., 2006; N. Smith et al., 2006; Dong et al., 2013; Khajanchi et al., 2016). The borrelial genome lacks an annotated CRP or cAMP phosphodiesterase; therefore, it is unclear how *B. burgdorferi*-generated cAMP might modulate the pathogenesis-specific regulation (Casjens et al., 2000). A previous study confirmed the AC enzymatic activity of recombinant borrelial CyaB (Khajanchi et al., 2016). During infection studies with a transposon *cyaB* mutant strain, it was found that *cyaB* did not play a role in tick to mouse transmission or mammalian infectivity when examined qualitatively by culture outgrowth. A borrelial Tn-seq identified *cyaB* as contributing to resistance to oxidative stress (Ramsey et al., 2017). The overall function of the CyaB enzyme in *B. burgdorferi* signal transduction and virulence factor regulation remains unclear, especially in the absence of any detectable downstream effector molecules that would recognize cAMP.

Borrelial *cyaB* overlaps with an intragenic small RNA (sRNA) SR0623 at the 3′ end of the open reading frame (ORF) and extends into the neighboring *bb0722* gene (Popitsch et al., 2017). sRNAs can be arranged in the genome as antisense, 5′ and 3′ untranslated region (UTR), intergenic, or intragenic (Papenfort and Vogel, 2010; Gottesman and Storz, 2011; Babitzke et al., 2019). sRNAs have a broad range of function with the ability to regulate translation of target mRNA and degradation of target mRNA, act as a riboswitch, or bind to proteins either altering or sequestering their activity. Recent sRNA transcriptome studies identified over 1,000 putative borrelial sRNA that are regulated in response to temperature shift and nutrient stress (Popitsch et al., 2017; Drecktrah et al., 2018).

In this study, we investigated the role of *cyaB* and SR0623 in borrelial pathogenesis. Our findings indicate that *cyaB* influences mammalian virulence in part through regulation of the BosR–RpoN–Rrp2–RpoS pathway. The regulation of *cyaB* was specific to interactions with host cells, further suggesting that this AC is important for the mammalian cycle of pathogenesis and may be responsive to unique host-specific signals. CyaB, and possible cAMP signaling, has the potential to be an uncharacterized signaling and regulation pathway important for the progression of Lyme disease.

## Materials and Methods

### Growth Conditions and Media

*Escherichia coli* was grown in Luria–Bertani (LB) broth supplemented with antibiotics at the following concentrations: kanamycin 50 μg/ml, spectinomycin 100 μg/ml, or gentamicin 15 μg/ml. *B. burgdorferi* was grown in Barbour–Stoener–Kelly II (BSKII) medium supplemented with 6% normal rabbit serum (NRS) under microaerophilic conditions at 32°C with 1% CO_2_ unless otherwise stated (Barbour, 1984). Modified BSK lacks bovine serum albumin (BSA), pyruvate, and NRS (Ramsey et al., 2017). BSK-lite was made using CMRL 1066 without l-glutamine and without glucose (USBiological) supplemented with 6% NRS and 0.01% L-glutamine (von Lackum and Stevenson, 2005). BSK-glycerol is BSK-lite with 0.6% glycerol. BSK media was supplemented with antibiotics at the following concentrations: kanamycin 300 μg/ml, streptomycin 100 μg/ml, or gentamicin 50 μg/ml.

### Plasmid Construction and Strain Generation

**FIGURE 1:**
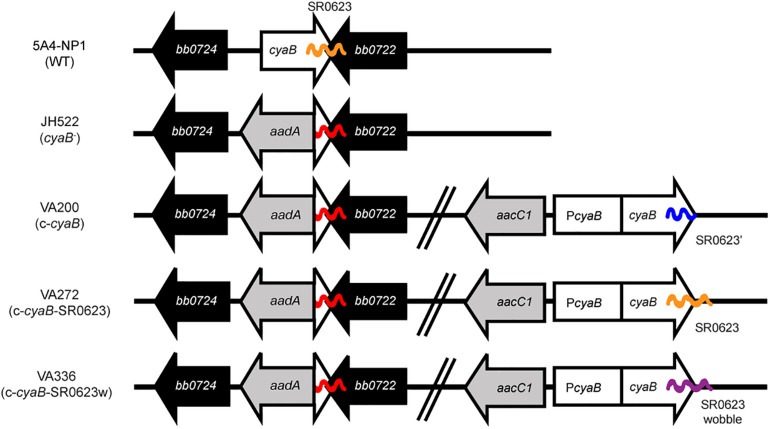
Schematic of the *cyaB* mutant and *trans*-complement strains. The *cyaB* deletion strain JH522 was generated through the insertion of an *aadA* antibiotic cassette to disrupt *cyaB* (*bb0723*) while keeping *bb0722* intact. Chromosomal *cyaB* complementation strains were made through the introduction of an *aacC1* antibiotic cassette. Complement strain VA200 contains the *cyaB* open reading frame (ORF) while truncating the sRNA SR0623, strain VA272 contains both the *cyaB* ORF and SR0623, and strain VA336 contains the *cyaB* ORF and a wobble mutation of SR0623. ORFs are indicated by arrows and sRNAs by wavy lines.

Strains, plasmids, and primers generated in this study are listed in Table 1 and Supplementary Table 1, respectively. The *cyaB* (*bb0723*) deletion construct was generated by amplifying upstream and downstream regions of approximately 1.5 kb and individually TOPO cloned into pCR8 (Thermo Fisher) resulting in pJH380 and pJH381, respectively. A *Kpn*I and *Bam*I digest inserted the downstream region from pJH381 into pJH380 to generate pJH383. The P*_*flgB*_-aadA* was PCR amplified and cloned into pCR2.1, designated pJH431, and cloned into pJH383 by *Sph*I and *Kpn*I digest generating the final deletion construct, pJH432. This final construct was transformed into *B. burgdorferi* ML23 and 5A4-NP1, resulting in JH441 and JH522, respectively (Figure 1; Labandeira-Rey and Skare, 2001; Lawrenz et al., 2002). A chromosomal *cyaB* complement construct, pJH446, was generated in the pJH333 backbone that encodes 1.5 kb chromosomal regions to allow allelic exchange between *bb0445* and *bb0446*, using P*_*flgB*_-aacC1* as the antibiotic selection (Li et al., 2007; Hyde et al., 2011b). JH441 was transformed with pJH446 resulting in strain JH446. The mutant and chromosomal complement strains were transformed with pBBE22*luc* to introduce constitutively expressed bioluminescence (Blevins et al., 2007; Hyde et al., 2011a).

**TABLE 1:** Strains and plasmids used in this study.

Strain	Genotype	References
***B. burgdorferi* strains**		
5A4-NP1	Clonal infectious isolate of 5A4 with *bbe02* disrupted with P*_*flgB*_-kan*, lacking cp9	Kawabata et al., 2004
JH522	5A4-NP1 *bb0723*:Sm^*R*^	This study
VA200	JH522 P*_*cyaB*_*512-FLAG-*cyaB*:Gent^*R*^	This study
VA272	JH522 P*_*cyaB*_*512-FLAG-*cyaB*-SR0623:Gent^*R*^	This study
VA336	JH522 P*_*cyaB*_*512-FLAG-*cyaB*-SR0623wobble:Gent^*R*^	This study
JH522 pVA102	JH522 carrying pVA87:P*_*pQE30*_*-FLAG-*cyaB*, Gent^*R*^	This study
ML23	Clonal isolate of strain B31 lacking lp25 and cp9	Labandeira-Rey and Skare, 2001
JH441 pBBE22*luc*	ML23 *bb0723*:Sm^*R*^ carrying constitutive bioluminescence shuttle vector	This study
JH446 pBBE22*luc*	JH441 P*_*cyaB*_*336*-cyaB-*SR0623*:*Gent^*R*^ carrying constitutive bioluminescence shuttle vector	This study
***E. coli* strains**		
NEB 10β	*araD139*Δ*(ara,leu)7697 fhuA lacX74 galK16 galE15 mcrA*φ*80d(lacZ*Δ*M15)recA1 relA1 endA1 nupG rpsL rph spoT1*Δ*(mrrhsdRMS-mcrBC)*	New England Biolabs
**Plasmids**		
pCR8	Intermediate for TOPO cloning, Spec^*R*^	Thermo Fisher
pENTR1a-N3xFLAG	Kan^*R*^	Thermo Fisher
pJH333	Allelic exchange vector, Spec^*R*^ and Gent^*R*^	This study
pVA110	Allelic exchange vector, Spec^*R*^ and Gent^*R*^	This study
pBSV2G	Shuttle vector, Gent^*R*^	Elias et al., 2003
pJSB268	pKFSS1:P*_*pQE30*_-luc* + P*_*flaB*_-lacI*, Spec^*R*^	Blevins et al., 2007
pJH380	pCR8 encoding 1.5 kb *cyaB* upstream region	This study
pJH381	pCR8 encoding 1.5 kb *cyaB* downstream region	This study
pJH383	pCR8 *cyaB* upstream and downstream regions	This study
pJH431	pCR2.1:P*_*flgB*_-aadA*	This study
pJH432	*cyaB* deletion construct, Spec^*R*^	This study
pJH446	pJH333:*cyaB*, Spec^*R*^	This study
pVA85	pENTR1a-N3xFLAG:*cyaB*-SR0623, Kan^*R*^	This study
pVA87	pJSB268 with Gent cassette, Gent^*R*^	This study
pVA102	pVA87:P*_*pQE30*_*-FLAG-*cyaB*, Gent^*R*^	This study
pVA112	pVA110:P*_*cyaB*_*512-FLAG-*cyaB*, Gent^*R*^	This study
pVA114	pVA110:P*_*cyaB*_*512-FLAG-*cyaB*-SR0623, Gent^*R*^	This study
pVA146	pVA110:P*_*cyaB*_*512-*cyaB*-SR0623wobble, Gent^*R*^	This study
pBBE22*luc*	Shuttle luminescent vector P*_*flaB*_*-Bb*luc*, Kan^*R*^	Hyde et al., 2011a
pCR2.1::βactin	pCR2.1 carrying murine β-actin, Kan^*R*^	Maruskova et al., 2008
pCR2.1::*recA*	pCR2.1 carrying *B. burgdorferi recA* Kan^*R*^	Wu et al., 2011

A similar construct, pVA110, was generated by amplifying the upstream *bb0445* fragment and P*_*flgB*_-aacC1* using primers *bb0445*-F-*Bam*HI/*bb0445*-R and P*flgB*-F-*Not*I/gent-R, respectively, and underwent overlap PCR with *bb0445*-F-*Bam*HI and P*flgB*-F-*Not*I, digested with *Bam*HI and *Not*I, and cloned into pJH333. The *cyaB* complement fragment *cyaB* with SR0623 was PCR amplified using the indicated primers in Supplementary Table 1, digested with *Not*I and *Xho*I, and ligated into pENTR1a-N3xFLAG (Thermo Fisher) to create plasmid pVA85. The complement fragment *cyaB* ORF was PCR amplified using primers P*cyaB*-F-*Sal*I/P*cyaB*-R and *cyaB*ORF-F/*cyaB*ORF-R-*Sal*I with pVA85 as the template and underwent overlap PCR with P*cyaB*-F-*Sal*I and *cyaB*ORF-R-*Sal*I. The complement fragment *cyaB* with SR0623 was amplified using the primer pair P*cyaB*-F-*Sal*I/P*cyaB*-R and *cyaB*ORF-F/*cyaB*SR0623-R-*Sal*I with pVA85 as the template and underwent overlap PCR with P*cyaB*-F-*Sal*I and *cyaB*SR0623-R-*Sal*I. The modified SR0623 sequence was engineered and manufactured by GenScript to alter the wobble base pair throughout the sRNA (Supplementary Figure 1). The complement fragments were cloned into pVA110 using the *Sal*I restriction sites resulting in pVA112, pVA114, and pVA146, respectively. Chromosomal complement plasmids were transformed into JH522 generating VA200, VA272, and VA336 (Figure 1 and Table 1).

To make a *trans* inducible FLAG-tagged *cyaB* complement with gentamicin resistance for overproduction of CyaB, the *Nde*I site from P*_*flgB*_-aacCI* was removed by amplifying the P*_*flgB*_* and the *aacC1* cassette with pBSV2G as the template. An overlap PCR was performed on the P*_*flgB*_* and the *aacC1*PCR products, digested, and ligated into pJSB268 digested with *Aat*II and *Bgl*II and blunt ended by Klenow (New England Biolabs) to create plasmid pVA87. To generate an N-terminally FLAG-tagged *cyaB* construct (pVA102), *cyaB* was amplified from pVA85, digested with *Nde*I and *Hin*dIII, and ligated into pVA87. JH522 was transformed with pVA102.

Electroporation of plasmid DNA into *B. burgdorferi* was done as previously described (Samuels et al., 1994; Hyde et al., 2011b). Up to 60 μg of DNA was transformed, recovered overnight, and then selected for by limiting dilution liquid plating in the appropriate antibiotic and 0.5% phenol red. Transformants were PCR screened for both the allelic exchange and plasmid content (Labandeira-Rey and Skare, 2001).

### Oxidative Stress Assays

Sensitivity to the oxidative stressor H_2_O_2_ was determined as previously performed (Hyde et al., 2009; Ramsey et al., 2017). Briefly, *B. burgdorferi* was grown in BSK-glycerol to mid-log phase, pelleted at 4,800 × *g* for 10 min at 4°C, washed with 1 × phosphate buffered saline (PBS), and resuspended in modified BSK; 5 × 10^7^ cells were treated with or without H_2_O_2_ and incubated at 32°C 1% CO_2_ for 4 h in a 1-ml volume. Samples were centrifuged at 6,600 × *g* for 10 min at 4°C, resuspended in BSKII with 6% NRS and 0.6% phenol red, serial diluted in 96-well plates, and incubated for 14 days to assess media color change and survival. Survival was measured from three biological replicates and data were converted to logarithmic values before calculating the averages.

### *B. burgdorferi* Co-Cultivation Assays

*Borrelia burgdorferi* was co-incubated with *Ixodes scapularis* embryonic cell line ISE6 and human neuroglioma cell line H4 (ATCC HTB-148) to evaluate bacterial transcriptional changes (Oliver et al., 1993; Schmit et al., 2011). ISE6 was maintained in L15C300 supplemented media at 34°C 2% CO_2_ in 25 cm^2^ flasks seeded with 1 × 10^7^ cells (90% confluency) for 24 h (J. D. Oliver et al., 2015). H4 cells were seeded at 90% confluency in Dulbecco’s modified Eagle’s medium (DMEM) (Sigma) supplemented with 10% FetalPlex (GeminiBio), herein designated DMEM + , at 37°C 5% CO_2_ for 17 h. *B. burgdorferi* was grown to mid-log phase under the same conditions as the cell line, and cells were pelleted, washed in PBS, and resuspended in cell culture media. *B. burgdorferi* were added to the ISE6 and H4 cells at a multiplicity of infection (MOI) of 10 and 40, respectively (J. H. Oliver et al., 1993; Livengood et al., 2008). Equivalent numbers of borrelial cells were incubated in cell culture media alone as a control. At 3, 6, and 24 h after infection, the cultivation media was collected for RNA isolation and qRT-PCR analysis.

### Western Blot Analysis

*Borrelia burgdorferi* were grown in BSK-lite or BSK-glycerol to mid-log phase, cell lysates were resolved on a 12.5% sodium dodecyl sulfate–polyacrylamide gel electrophoresis (SDS-PAGE) and transferred to a polyvinylidene difluoride (PVDF) membrane, and western immunoblotting was conducted as previously described (Labandeira-Rey and Skare, 2001; Saputra et al., 2020). The antibody was generated in Sprague–Dawley rats against PlzA as previously described (Miller et al., 2016; Izac et al., 2019). The following primary antibody concentrations were used: mouse anti-flagellum (1:4,000) (Affinity Bioreagent), mouse anti-FLAG (1:4,000) (Sigma), rabbit anti-P66 (1:5,000) (Cugini et al., 2003), rabbit anti-BosR (1:1,000) (Seshu et al., 2004b), rabbit anti-DbpA (1:10,000) (Guo et al., 1998), rat anti-PlzA (1:1,000), mouse anti-BadR (1:1,000) (Miller et al., 2013), mouse anti-OspC (1:20,000) (He et al., 2014), mouse-anti-OspA (1:1,000) (Capricorn), and mouse anti-Rrp2 (1:1,000) (Yang et al., 2003). Secondary antibodies were coupled to horseradish peroxidase (HRP): donkey-anti-rabbit IgG HRP (Amersham), goat-anti-mouse IgG HRP (Thermo Fisher), and rabbit-anti-rat IgG HRP (Thermo Fisher). Membranes were imaged with chemiluminescent substrates to detect antigen–antibody complexes. The immunoblot data presented are representative of at least three biological replicates.

### Reverse Transcriptase PCR and Quantitative RT-PCR

*Borrelia burgdorferi* RNA was isolated using hot phenol chloroform extraction as previously described (Lybecker et al., 2014; Lybecker and Henderson, 2018). Total RNA was treated with DNaseI (Roche) and 1 μg was converted to cDNA using SuperScript III reverse transcriptase (+ RT) (Thermo Fisher) according to the manufacturer’s instructions. A no RT control was included for each RNA sample. PCR reactions using 500 ng cDNA as template were amplified with AccuStart II PCR Supermix (Quantabio) and imaged on a 1% agarose gel. Quantitative RT-PCR (qRT-PCR) reactions were performed from *in vitro* cultivated samples with 50 ng + RT and −RT cDNA using a ViiA 7 Real-Time PCR system (Applied Biosystems) and Fast SYBR Green Master Mix (Applied Biosystems) according to the manufacturer’s instructions. *B. burgdorferi* co-culture transcript experiments were performed using PerfeCTa SYBR Green FastMix ROX (Quantabio) and StepOnePlus Real-Time PCR system (Applied Biosystems). *flaB* was used as an internal control and fold change relative to wild type (WT) calculated using the 2^–ΔΔ*CT*^ method from three to four biological and technical replicates (Livak and Schmittgen, 2001).

### Northern Blots

RNA was collected from *B. burgdorferi* strains grown in BSK-glycerol to mid-log phase at 32°C 1% CO_2_. RNA isolation and Northern blot analysis were performed as previously described (Popitsch et al., 2017). Seven to 10 μg of RNA was denatured in 2 × RNA load dye (Thermo Fisher) and heated to 65°C for 15 min, loaded onto a Novex Pre-cast 6% TBE-Urea (8 M) polyacrylamide gel (Thermo Fisher) in 1 × TBE, and run for 45–60 min. RNA was electroblotted at room temperature (10 V for 1 h in 0.5 × TBE) to HybondXL membranes (Amersham). The membranes were UV cross-linked (Fisher Scientific UV Crosslinker FB-UVXL-1000) and probed with DNA oligonucleotide (Supplementary Table 1) in OligoHyb buffer (Thermo Fisher) per the manufacturer’s protocol. Oligonucleotide probes were end-labeled with γ-^32^P ATP (PerkinElmer) and T4 PNK (New England Biolabs) per the manufacturer’s instructions. Unincorporated P^32^ was removed using illustra^TM^ MicroSpin^TM^ G50 columns (GE Healthcare). Purified probes were heated at 95°C for 5 min before being added to the prehybridizing blots. Blots were hybridized at 42°C rotating overnight. Membranes were washed 2 × 30 min in wash buffer (2 × SSC 0.1% SDS). Membranes were placed on Kodiak BioMax maximum sensitivity (MS) autoradiography film and placed at –80°C for 1–10 days depending on the radiation emission given by each membrane. Film was developed on an AFP imaging developer and scanned using an Epson Expression 10000XL. 5S rRNA was used as the loading control. The Northern blot data presented are representative of three biological replicates.

### Mouse Infection Studies

Infection studies were conducted using 6–8-week-old C3H/HeN female mice (Charles Rivers) with 5A4-NP1, JH522, VA200, VA272, or VA336. Four to five mice were infected with 10^5^ *B. burgdorferi* by ventral intradermal (ID) injection. Mice were sacrificed and tissues aseptically collected at 7, 14, and 21 days postinfection (dpi) for cultivation or qPCR of borrelial load. Outgrowth of viable *B. burgdorferi* was determined by dark-field microscopy and the percent positive tissues was determined. Mice tissues were harvested and DNA was isolated using the DNeasy Blood and Tissue kit (Qiagen) according to the manufacturer’s instructions with the addition of 40 μl of 10% collagenase (Sigma) and incubated at 55°C overnight. qPCR reactions were performed using a StepOnePlus Real-Time PCR system (Applied Biosystems) and PowerUp SYBR Green Master Mix (Applied Biosystems) according to the manufacturer’s instructions. Standard curves were used to determine the absolute quantification of mouse β-actin and *B. burgdorferi recA*. All standard curve reactions had an *R*^2^-value above 0.9. Technical triplicates were measured for each sample and values are displayed as copies of *B. burgdorferi recA* per 10^6^ mouse β-actin.

To spatially and temporally track luminescent *B. burgdorferi* during infection, an *in vivo* imaging system (IVIS) was used to image mice (IVIS Spectrum, PerkinElmer). IVIS infection studies were conducted using 6–8-week-old Balb/c female mice (Charles Rivers) as previously described (Hyde et al., 2011a; Hyde and Skare, 2018). Briefly, groups of five mice were ID infected with 10^5^ *B. burgdorferi* strain ML23 pBBE22*luc*, JH441 pBBE22*luc*, or JH446 pBBE22*luc*. Mice were intraperitoneally (IP) treated with 5 mg of D-luciferin and imaged at 1 h and 1, 4, 7, 10, 14, and 21 dpi. One infected mouse of each group did not receive D-luciferin to serve as a negative control for background luminescence. Images were collected with 1 and 10 min exposures and bioluminescence from the whole body was quantitated. Images in the 600–60,000 counts range were used to quantitate bioluminescence. Background bioluminescence was subtracted from the treated samples and averaged. Mice were sacrificed 21 dpi and harvested tissues were used for cultivation as described above.

### Statistical Analyses

Statistical analysis was performed using GraphPad Prism (GraphPad Software, Inc., La Jolla, CA, United States). The statistical analysis used is listed in the figure legends. Significance was determined by *p* values equal to or less than 0.05.

## Results

### Construction of the *cyaB* and SR0623 Mutant and Complement Strains

To investigate *B. burgdorferi cyaB*, we generated a deletion of *bb0723* by replacing the ORF with the P*_*flgB*_-aadA* antibiotic cassette in 5A4-NP1 (WT) resulting in strain JH522 (Figure 1 and Table 1) (Kawabata et al., 2004). The 3′ end of *cyaB* overlaps with the 3′ end of its neighboring gene *bb0722* by 26 base pairs, which was not deleted in the *cyaB*^–^ strain. In addition, the sRNA SR0623 is encoded within the 3′ end of *cyaB* (95 base pairs) and with *bb0722* (88 base pairs) (Popitsch et al., 2017). SR0623 could either be a result of RNA processing of the *cyaB* mRNA or it could have its own promoter within *cyaB*. Adams et al. globally identified the 5′ end transcriptome and identified putative transcriptional start sites (TSSs) in *B. burgdorferi* (Adams et al., 2017). A putative TSS was not identified within the *cyaB* ORF suggesting SR0623 is synthesized *via cyaB* mRNA processing. To distinguish the functional contribution of CyaB from the sRNA SR0623, we made three chromosomal complements of *cyaB* using its native promoter and P*_*flgB*_-aacC1* antibiotic cassette but included different forms of SR0623 (Figure 1). VA200 encodes *cyaB* and a truncated SR0623, designated c-*cyaB*. A *cyaB* and full-length SR0623 is restored in VA272 and named c-*cyaB*-SR0623. A site-directed mutant of SR0623 was generated by altering every third base pair in the wobble position to disrupt the sRNA primary and secondary structures while maintaining the amino acid sequence of CyaB in complement strain VA336, referred to as c-*cyaB*-SR0623w. For independent verification of the *cyaB* phenotype, deletion and complement strains were also generated in the ML23 background strain. The *cyaB*^–^ strain, JH441, was chromosomally complemented with *cyaB* and SR0623, generating the c-*cyaB*-SR0623 strain JH446. JH441 and JH446 were transformed with pBBE22*luc* for *in vivo* imaging studies (Hyde et al., 2011a; Hyde and Skare, 2018). There was no observable difference in growth rate between the WT and *cyaB*^–^ strains generated (data not shown).

**FIGURE 2:**
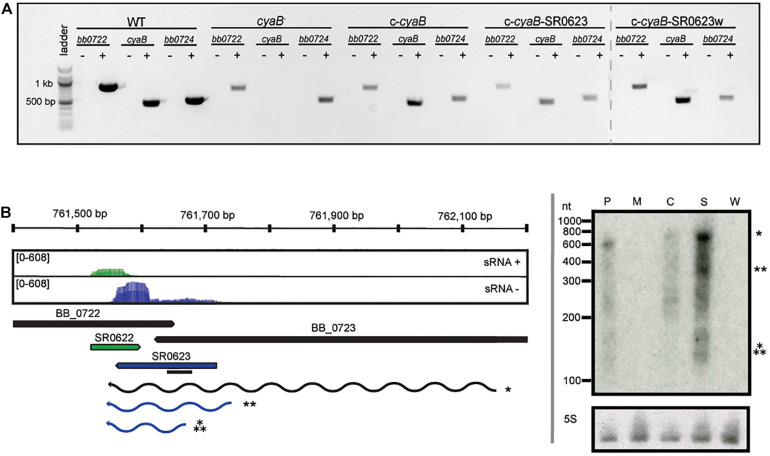
Verification of strains generated in this study. **(A)** Deletion of *cyaB* does not abolish transcription of neighboring genes *bb0722* or *bb0724*. RT-PCR was used to verify the presence or absence of transcripts in the indicated *B. burgdorferi* strains. RNA was isolated and used to generate cDNA as describe in the *Methods*. The ± symbols indicate the cDNA reaction with or without reverse transcriptase. PCR reactions included cDNA and primers for *cyaB* (*bb0723*), *bb0722*, or *bb0724*. **(B)** sRNA-sequencing results are displayed in the coverage map (Popitsch et al., 2017). The + strand is shown in green and the − strand in blue. Northern blot analyses of total RNA fractionated on a 6% denaturing polyacrylamide gel, blotted to a nylon membrane, and hybridized with radioactive oligonucleotides. The black line represents the location of the oligonucleotide probes. The genomic context is indicated below the coverage plot. The predicted transcripts are denoted and marked with the appropriate band in the Northern blot. Northern blots are representative of three biological replicates. The following abbreviations are used to indicate strains: WT (P), *cyaB*^–^ (M), c-*cyaB* (C), c-*cyaB*-SR0623 (S), and c-*cyaB*-SR0623w (W).

The absence of polar effects on the neighboring genes and confirmation of *cyaB* expression in our strains were verified qualitatively by reverse transcriptase PCR (RT-PCR) (Figure 2A). As expected, *cyaB* transcript was detected in WT, c-*cyaB*, c-*cyaB*-SR0623, and c-*cyaB*-SR0623w with a notable absence in the *cyaB*^–^ strain. Expression of *bb0722* and *bb0724* was observed in all strains; however, we cannot rule out the possibility that there are quantitative differences in the expression levels. To evaluate the relative steady-state levels and transcript lengths of *cyaB* and SR0623, Northern blots were performed with probes designed to hybridize to the 5′ end of SR0623 (Figure 2B). The *cyaB* transcript (484 bp) and SR0623 (∼158 bp) are present in the WT strain and absent in the *cyaB*^–^ strains, as anticipated. c-*cyaB* produces a *cyaB* transcript and lacks SR0623. c-*cyaB*-SR0623 expresses more *cyaB* and SR0623 than the WT strain, which may alter the levels of CyaB protein and perhaps the function of SR0623. c-*cyaB*-SR0623w strain does not have a detectable *cyaB* or SR0623 because the engineered wobble-base mutations prevent the probe from binding. Together, these data suggest that steady-state levels of the *cyaB* mRNA are dependent on its 3′ UTR, which also encodes SR0623.

### *cyaB* Does Not Contribute to the Oxidative Stress Response

**FIGURE 3:**
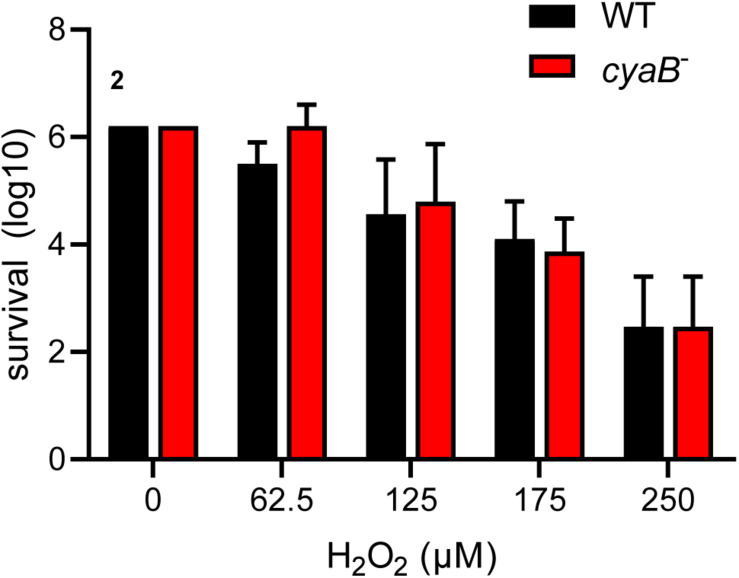
*cyaB* does not contribute to H_2_O_2_ resistance. WT and *cyaB*^–^ strains were grown in BSK-glycerol to mid-log phase at 32°C 1% CO_2_, exposed to H_2_O_2_ in modified BSK media for 4 h at 32°C 1% CO_2_, and serial diluted for outgrowth to determine survival. Shown are the average and standard error of three independent biological replicates.

*Borrelia burgdorferi* is able to sense and combat oxidative stress by mechanisms that are not fully understood (Boylan et al., 2003,BR10,BR11; Seshu et al., 2004b; Hyde et al., 2009; Ramsey et al., 2017). A Tn-seq screen by Ramsey et al. found that *B. burgdorferi* disrupted in *cyaB* had a two-fold decrease in fitness after exposure to H_2_O_2_; therefore, we sought out to determine if the AC contributed to the oxidative stress response similar to other pathogens (Ramsey et al., 2017). *B. burgdorferi* strains, WT and *cyaB*^–^, were exposed to increasing concentrations of H_2_O_2_ and then serial diluted to determine the survival percentage. We found that *cyaB*^–^ strain survival was comparable to the WT strain 5A4-NP1 (Figure 3). There are several reasons our *cyaB*^–^ phenotype does not correlate with the *cyaB* Tn-seq phenotype of Ramsey et al. These authors exposed a pool of transposon disruption mutants to an oxidative stressor and measured the fitness of the population, whereas we examined a single strain and its survival. Another reason for the difference in findings may be the *B. burgdorferi* growth condition prior to conducting the assay. We grew our cells to mid-log phase in BSK-lite lacking glucose and supplemented with glycerol rather than complete BSKII. It was important for us to limit glucose in the media because in some bacteria, such as *Vibrio cholerae* and *E. coli*, the presence of glucose has been shown to alter AC activity and, therefore, cAMP production (Gstrein-Reider and Schweiger, 1982; Liang et al., 2007). Previous studies used BSK-glycerol to grow *B. burgdorferi* when investigation of secondary metabolite c-di-GMP was evaluated (He et al., 2011). We found that the absence or addition of glycerol to the BSK-lite did not influence *B. burgdorferi* growth or alter mammalian virulence factor production (Supplementary Figure 2).

### *cyaB* Influences Borrelial Virulence Determinants

**FIGURE 4:**
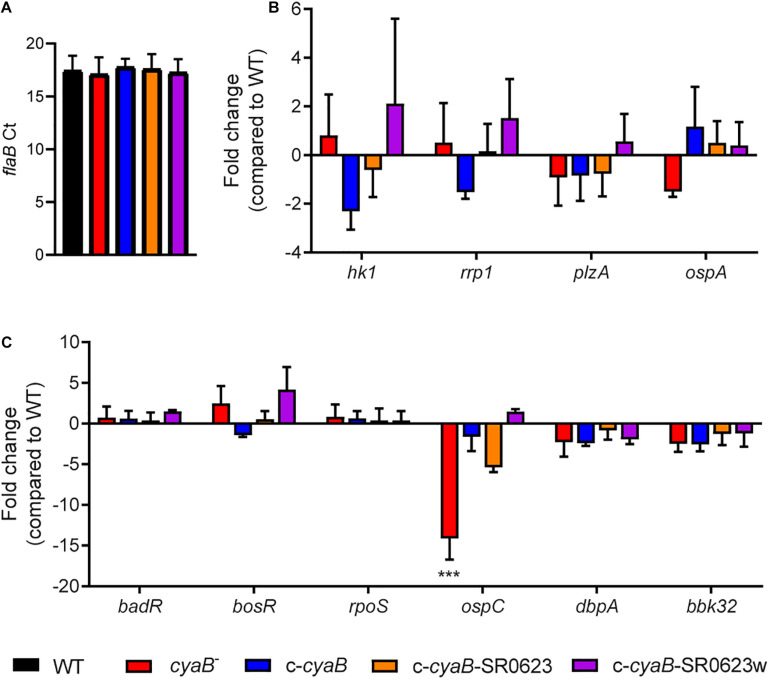
Deletion of *cyaB* reduces *ospC* expression. *B. burgdorferi* was grown in BSK-glycerol to mid-logarithmic phase at 32°C 1% CO_2_ for qRT-PCR analysis. **(A)** Comparison of the Ct values for the endogenous reference gene *flaB*. Relative transcript levels of virulence determinants for the **(B)** tick and **(C)** mammalian cycle. Fold changes are compared to WT. Shown are the averages and standard error of three biological replicates. Statistical analysis was performed using one-way ANOVA with Dunnett correction relative to WT, ***p < 0.001.

Loss of an AC results in attenuation of virulence factors in many different bacteria, such as *Pseudomonas aeruginosa* and *Salmonella typhimurium*; therefore, we were interested in examining the influence of *cyaB* on important *B. burgdorferi* virulence determinants (Curtiss and Kelly, 1987; R. S. Smith et al., 2004). Transcript levels were measured for multiple *B. burgdorferi* targets that included genes important for regulation in the tick vector (*hk1*, *rrp1*, *plzA*, and *ospA*) and mammalian virulence genes (*badR*, *bosR*, *rpoS*, *ospC*, *dbpA*, and *bbk32*) (Figures 4B,C). No changes were observed for genes shown to be operative in the arthropod-borne phase of infection, which may be due to secondary messenger c-di-GMP being involved in the vector (Figure 4B; Rogers et al., 2009; He et al., 2014). Surprisingly, only *ospC* transcription was found to be statistically significantly reduced 14-fold in the *cyaB*^–^ strain compared with WT (Figure 4C). The expression of *ospC* was fully and partially restored to WT level in the c-*cyaB*-SR0623w and c-*cyaB* strains, respectively. The c-*cyaB*-SR0623 had an *ospC* transcript level similar to the *cyaB*^–^ strain. These transcriptional data indicate that *cyaB* may be able to influence *B. burgdorferi* mammalian virulence determinant expression.

**FIGURE 5:**
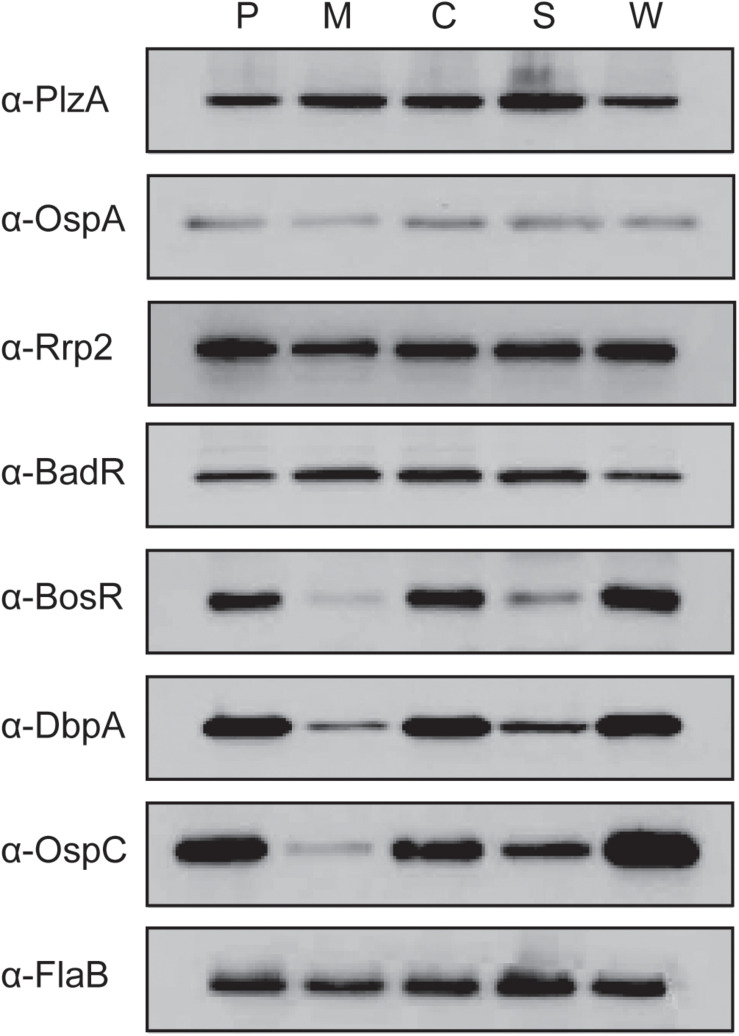
Deletion of *cyaB* reduces protein production of BosR, OspC, and DbpA. *Borrelia burgdorferi* was grown in BSK-glycerol to mid-logarithmic phase at 32°C 1% CO_2_. Protein was harvested and resolved on SDS–PAGE with approximately 4 × 10^7^ *B. burgdorferi* in each lane. Immunoblots were prepared using the depicted antiserum. FlaB was used as a loading control. Displayed is the representative of three independent replicates. The following abbreviations are used to indicate strains: WT (P), *cyaB*^–^ (M), c-*cyaB* (C), c-*cyaB*-SR0623 (S), and c-*cyaB*-SR0623w (W).

Cyclic adenosine monophosphate plays an important role in posttranscriptional regulation in many bacteria; for example, in *Salmonella enterica*, cAMP-CRP posttranscriptionally regulates transcriptional regulator HilD resulting in reduced virulence factor production (El Mouali et al., 2018). Our next step was to evaluate the impact of *cyaB* on the protein production of *B. burgdorferi* virulence determinants. We used immunoblotting to examine the protein levels of several borrelial components associated with the tick and mammalian pathways (Figure 5). Virulence determinants PlzA and OspA, important for survival in the tick, and mammalian virulence determinants Rrp2 and BadR were not altered in the *cyaB*^–^ strain compared with WT. *bosR* undergoes transcriptional and posttranscriptional regulation in response to pH or metals and CO_2_, respectively, by unknown mechanisms; therefore, we considered that BosR may also be posttranscriptionally regulated by cAMP (Hyde et al., 2007; Saputra et al., 2020). We found that the *cyaB*^–^ strain produced less BosR relative to WT demonstrating another condition where BosR is posttranscriptionally regulated given there was no difference observed in *bosR* expression (Figures 4, 5). Strains c-*cyaB* and c-*cyaB*-SR0623w restored BosR protein production back to approximate WT levels. However, the c-*cyaB*-SR0623 complement had BosR protein levels comparable to the *cyaB*^–^ strain. The lack of complementation of c-*cyaB*-SR0623 may be in part due to different steady-state levels of *cyaB* and SR0623 observed by Northern blot analysis (Figure 2B). DbpA and OspC protein production followed the same pattern as BosR. RpoS protein levels were also investigated; however, under the growth conditions used, RpoS production was below the level of detection using Western immunoblots (data not shown). The different phenotypes of complement *cyaB* strains suggest a possible regulation of *cyaB* by SR0623. Collectively, these results would indicate that CyaB or cAMP, directly or indirectly, posttranscriptionally activates both BosR and DbpA. These data confirm that the changes observed in the *ospC* transcript influence the OspC protein level. It remains unclear if OspC and DbpA are being regulated by *cyaB* independently or through BosR regulation of *rpoS*.

### *cyaB* Expression is Induced by Host Cell Interaction

**FIGURE 6:**
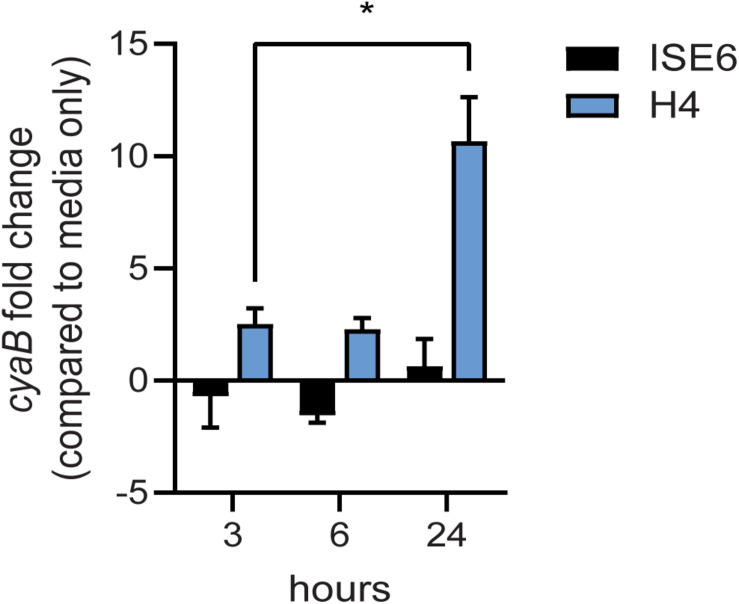
*cyaB* expression is induced with mammalian H4 cell co-culture. *Borrelia burgdorferi* was co-cultured with ISE6 tick cells or H4 mammalian cells. qRT-PCR was performed on samples collected at 3, 6, and 24 h co-incubation. *flaB* was used as an endogenous control. Shown is the average and standard error of four biological replicates. Statistical analysis was done using two-way ANOVA with Tukey correction, *p-value < 0.05.

*Borrelia burgdorferi* gene expression is highly responsive to changes in various environmental cues and may also impact *cyaB* expression. Under BSK cultivation with shifts in temperature, pH, and CO_2_, we observed no significant changes in *cyaB* transcript (data not shown). To investigate if expression of *cyaB* is influenced by tick or mammalian cellular factors, we co-cultured *B. burgdorferi* strain 5A4-NP1 for 24 h with the tick neuroglial cell line ISE6 or the mammalian neuroglial cell line H4 and harvested bacteria in the cell culture media (Figure 6; Oliver et al., 1993; Livengood et al., 2008). *cyaB* expression was not significantly changed at the time points tested in the tick neuroglial cell line ISE6 relative to *B. burgdorferi* incubated in cell culture media alone. Mammalian neuroglial cell line H4 significantly induced the borrelial *cyaB* expression after 24 h of co-incubation. These results indicate that *cyaB* expression is influenced by cellular factor(s) unique to mammalian cell lines and not by *in vitro*-grown tick cells or during *in vitro* cultivation in BSKII media.

### The Absence of *cyaB* Results in Attenuated Infection

**FIGURE 7:**
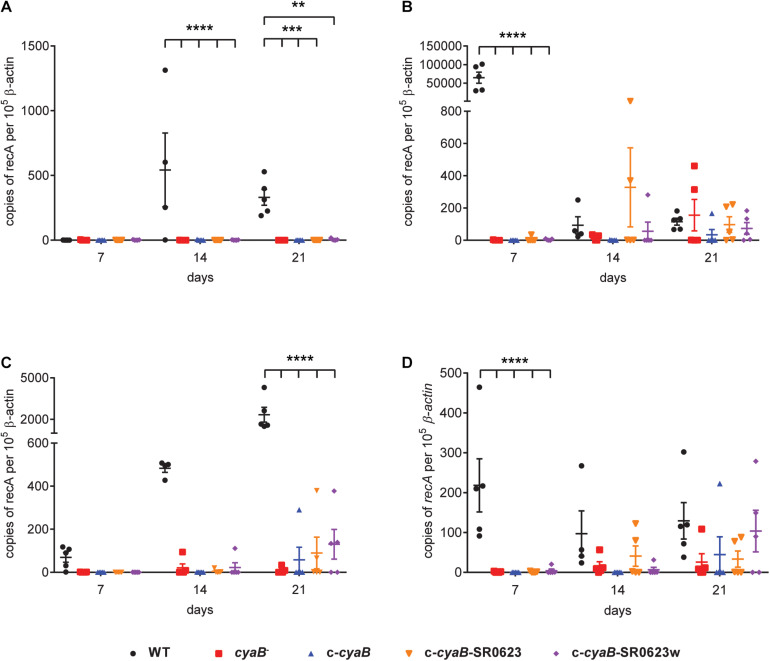
Borrelial burden and tissue dissemination is reduced in mouse tissues infected with the *cyaB* mutant. C3H/HeN mice were infected with 10^5^ *Borrelia burgdorferi* and tissues were harvested at 7, 14, and 21 dpi. qPCR was performed on **(A)** ears, **(B)** skin flanks, **(C)** joints, and **(D)** bladders to determine the number of borrelial genomes (*recA*) per 10^6^ copies of mouse β-actin. Individual data points with at least *n* of 4 with lines representing average and standard error. Statistical analysis was done using two-way ANOVA with Dunnett correction relative to WT, **p-value < 0.01, ***p-value < 0.001, ****p-value < 0.0001.

Given that deletion of *cyaB* resulted in altered mammalian virulence determinants, we hypothesized that infecting mice with the *cyaB*^–^ strain would alter infectivity. C3H/HeN mice were intradermally infected with the WT, *cyaB*^–^ strain, or complement strains. Tissues were collected at 7, 14, and 21 dpi for both qualitative outgrowth and quantitative molecular analysis of infection. Cultivation data show that all harvested tissues from mice infected with the WT strain were positive for *B. burgdorferi* by day 14, whereas half the tissues for the *cyaB*^–^ strain did not have bacterial outgrowth (Table 2). Unfortunately, none of the three complement strains were able to restore infectivity to WT levels. This was surprising given that the qRT-PCR, Northern, and Western data show that the c-*cyaB* and c-*cyaB*-SR0623w strains restore the *cyaB in vitro* deletion phenotypes. *B. burgdorferi* burden of individual infected tissues was analyzed by qPCR to determine the bacterial burden. The *cyaB*^–^ strain was significantly lower at 7 dpi in bladders and skin flanks adjacent to the inoculum site. The ears, a distal skin colonization, and joints had less *B. burgdorferi* at 14 and 21 dpi, respectively, when infected with the *cyaB*^–^ strain (Figure 7). The *cyaB* complements had borrelial loads similar to the *cyaB*^–^ strain and, therefore, did not restore infectivity. Taken together, these data would indicate that *cyaB* plays a role in mammalian infectivity.

**TABLE 2:** Tissue infectivity of *B. burgdorferi*-infected mice.

Strain	Number of positive cultures/total				
	Lymph node	Skin flank	Ear	All sites	% positive all sites
**Day 7**					
WT	5/5	5/5	1/5	11/15	73
*cyaB*^–^	0/5	1/5	0/5	1/15	6
c-*cyaB*	0/5	0/5	0/5	0/15	0
c-*cyaB*-SR0623	1/5	1/5	0/5	2/15	13
c-*cyaB*-SR0623w	2/5	4/5	0/5	6/15	40
**Day 14**					
WT	4/4	4/4	4/4	12/12	100
*cyaB*^–^	3/5	2/5	0/5	5/15	33
c-*cyaB*	0/5	0/5	0/5	0/20	0
c-*cyaB*-SR0623	2/5	2/5	0/5	4/15	26
c-*cyaB*-SR0623w	1/5	1/5	1/5	3/15	20
**Day 21**					
WT	5/5	5/5	5/5	15/15	100
*cyaB*^–^	2/5	2/5	0/5	4/15	26
c-*cyaB*	1/5	1/5	0/5	2/15	13
c-*cyaB*-SR0623	3/5	3/5	0/5	6/15	40
c-*cyaB*-SR0623w	3/5	3/5	1/5	7/15	46

**FIGURE 8:**
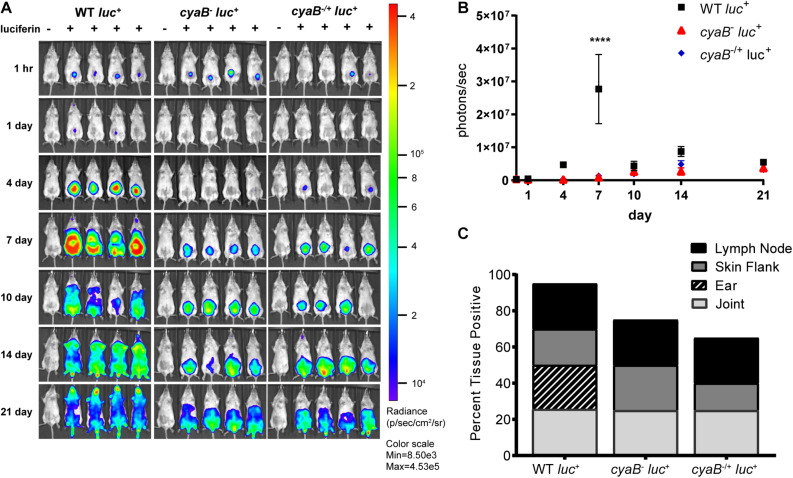
Bioluminescent *cyaB*^–^ has attenuated infection and dissemination. **(A)** Bioluminescent *Borrelia burgdorferi* is temporal and spatial tracked during infection of Balb/c mice with 10^5^ WT, *cyaB*^–^, or c-*cyaB*-SR0623. Mice were imaged at 1 h and 1, 4, 7, 10, 14, and 21 dpi. The mouse in the first position of the image, indicated by (-), did not receive D-luciferin to serve as a background control. *n* of 5 for each infection group. **(B)** The bioluminescence of four mice was quantitated and averaged. Statistical analysis was performed using two-way ANOVA with Tukey correction relative to WT, ****p-value < 0.0001. **(C)** The percentage of tissues positive for *B. burgdorferi* at 21 dpi, grown in BSKII + NRS.

To independently evaluate the influence of *cyaB* on infectivity, we generated a *cyaB* deletion in the ML23 background that is used to track bioluminescent imaging during murine infection as a way to evaluate temporal and spatial dissemination (Hyde et al., 2011a, 32; Hyde and Skare, 2018). The parent (ML23), *cyaB*^–^ (JH441), and c-*cyaB-*SR0623 complement (JH446) strains had pBBE22*luc* introduced into them and were tested for disseminated infectivity using light emission as a reporter for live *B. burgdorferi* (Hyde et al., 2011a). Western analysis of the aforementioned showed less protein production of BosR, DbpA, and OspC in the *cyaB*^–^ strain as was observed in the 5A4-NP1 background mutant (Supplementary Figure 3). Balb/c mice were then infected with the *B. burgdorferi* strains and *in vivo* imaged at 0, 1, 4, 7, 10, 14, and 21 dpi. The bioluminescent tracking shows the bacteria being localized to the site of injection early at day 0 and then progressing to distal tissues throughout the mouse by day 21 (Figure 8A). At 7 dpi, the peak of infection, the parent strain produces three times more light than the *cyaB*^–^ strain; however, the c-*cyaB-*SR0623 complement strain is not able to restore the light emission observed for the parent strain (Figure 8B). The parent strain disseminates to distal tissues through day 21. This dissemination is not observed in the *cyaB*^–^ or c-*cyaB-*SR0623 strains, which instead stay localized near the site of injection. After imaging on day 21, mice tissues, lymph nodes, skin flanks, ears, and joints were harvested and used for cultivation. We found a slight reduction in the number of infected tissues in the *cyaB*^–^ strain and c-*cyaB-*SR0623 *B. burgdorferi* (Figure 8C). It is interesting to note that the ears of the *cyaB*^–^ strain and c-*cyaB*-SR0623 strain infected mice were negative for *B. burgdorferi*, suggesting the genetic modifications may alter tissue dissemination. This independently validates the infectivity data in the 5A4-NP1 background and, taken together, despite the issues with incomplete complementation, supports the finding that *cyaB* is important for murine infection.

## Discussion

*Borrelia burgdorferi* gene regulation is dynamic and highly responsive to changes in environmental conditions to support the necessary adaptation for traversing between the tick vector and mammalian host (Radolf et al., 2012; D. Scott Samuels and Samuels, 2016). The mechanisms used by *B. burgdorferi* to directly sense environmental conditions and relay that information to alter gene regulation are poorly understood. We hypothesize that *B. burgdorferi* uses an AC (*cyaB*, *bb0723*) and possibly cAMP for the response to environmental cues and to regulate virulence determinants important for mammalian infection. ACs cyclize ATP producing cAMP which functions as a secondary messenger in eukaryotes and prokaryotes (Stanley McKnight, 1991; Botsford and Harman, 1992; Kamenetsky et al., 2006; McDonough and Rodriguez, 2011). In bacteria, ACs and/or cAMP is responsive to a variety of environmental changes, such as carbon starvation, CO_2_ levels, bicarbonate, osmolarity, and pH (Hoffmaster and Koehler, 1997; Cann et al., 2003; Franchini et al., 2015; Rebollo-Ramirez and Larrouy-Maumus, 2019). cAMP is used by numerous bacterial pathogens to alter both the host and pathogen at the level of posttranscriptional regulation for signal reception, signal transduction, AC activity, virulence gene regulation, resistance to oxidative stress, and persistence (Molina-Quiroz et al., 2018). It is well documented that *B. burgdorferi* utilizes cyclic dinucleotides during the tick and mammalian stages of pathogenesis to modulate the necessary gene regulation for its response to environmental pressures; therefore, it is plausible that it also relies on cyclic nucleotides for regulation (Rogers et al., 2009; He et al., 2011; Ye et al., 2014; Savage et al., 2015; Melissa J. Caimano et al., 2015; Zhang et al., 2018). It is important to understand the strategies employed by *B. burgdorferi* to adapt to changing environmental conditions to evaluate borrelial pathogenesis in the context of mammalian infection.

In this study, a genetic approach was used to evaluate the borrelial *cyaB* contribution to the regulation of virulence determinants and mammalian infectivity. Borrelial *cyaB* has been annotated as a class IV AC, which is the smallest of the classes and has been previously crystallized in *Yersinia pestis* (Casjens et al., 2000; Khajanchi et al., 2016). *cyaB* is encoded on the positive strand and overlaps with *bb0722* encoded on the opposite strand. Deletion mutants of *cyaB* in two independent *B. burgdorferi* strains, 5A4-NP1 and ML23, were generated that also disrupted SR0623. Three unique complement strains, *cyaB* only, *cyaB* with SR0623, and *cyaB* with a mutagenized SR0623, were generated to clarify the contribution of *cyaB*, SR0623, and the combination of *cyaB* and SR0623 to our readouts of borrelial infectivity (Figure 1). The complete deletion of the *cyaB* ORF also truncates SR0623 and results in a reduction in the production of important mammalian virulence determinants BosR, OspC, and DbpA, while tick virulence determinants are unchanged (Figure 5). Interestingly, only *ospC* was transcriptionally downregulated in the *cyaB* deletion strains (Figure 4C). Complement strains encoding the *cyaB* ORF with a truncated or mutagenized SR0623 were able to restore protein production to WT levels, suggesting that sRNA may not be necessary for the regulation of OspC, BosR, or DbpA. Unexpectedly, the complement with *cyaB* and a complete SR0623 produced only slightly higher levels of BosR, OspC, and DbpA than the mutant, alternatively leaving open the possibility that SR0623 could have regulatory effects beyond *cyaB* and may include borrelial virulence determinants. Northern analysis demonstrated higher relative abundance of *cyaB* transcript and SR0623 in the *cyaB*-SR0623 complement strain compared with WT, which may explain the partial complementation phenotype (Figure 2). These data demonstrate that *cyaB* contributes to transcriptional and posttranscriptional regulation of selected *B. burgdorferi* genes. Furthermore, *cyaB* and possibly cAMP are involved in regulation of factors specific for borrelial pathogenicity.

*Borrelia burgdorferi* is greatly influenced by environmental conditions and may use *cyaB* as an environmental sensor (Radolf et al., 2012; D. Scott Samuels and Samuels, 2016). We examined the *cyaB* mutant and complement strains under a variety of growth conditions by imposing oxidative stress, as well as shifting temperature, pH, and CO_2_, and found no phenotypic differences (Figure 3 and data not shown). Knowing that different carbon sources can alter cyclase activity and the production of cyclic nucleotide and di-nucleotides, media that replaced glucose with glycerol were used to examine the regulation of borrelial virulence factors (Peterkofsky and Gazdar, 1974; Liu et al., 2020). Differences in borrelial virulence determinant protein production were more pronounced in BSK-lite, independent of glycerol supplementation, relative to conventional BSKII (Supplementary Figure 2 and data not shown). Further investigation indicated that BSK-lite with or without glycerol resulted in the same pattern of protein production signifying that glucose as the carbon source was responsible for the differential response and demonstrated borrelial catabolite repression.

*Borrelia burgdorferi* is highly responsive to host-specific signals and it is possible that *cyaB* is involved in signaling for mammalian adaptation. We evaluated *cyaB* expression when co-cultured with vector or mammalian cells to mimic interactions during the pathogenic cycle. The expression of *cyaB* is induced when co-cultured with mammalian H4 neuroglial cells, but unchanged with tick ISE6 cells (Figure 6). Due to *cyaB*-induced expression in the presence of mammalian cells, we also evaluated the contribution of *cyaB* to mammalian infection. During mouse infection, the *cyaB* mutant strains had lower borrelial colonization, particularly during early time points (Figures 7, 8). In later infection, disseminated tissues, notably the ear and tibiotarsal joint, also had lower *B. burgdorferi* load relative to the infectious parent strain. Unfortunately, the complement strains that restored virulence determinant production *in vitro* did not colonize tissues at the same level as parental *B. burgdorferi* or restore bacterial burden. The data are strengthened by similar results in two independent borrelial strains. Infectivity studies demonstrated that the absence of *cyaB* results in inhibited dissemination and attenuated infection. Together, this suggests *cyaB* contributes to mammalian colonization and supports this stage of the life cycle.

Khajanchi et al. (2016) evaluated the functional ability of *cyaB* and its contribution to mouse infection using the *cyaB* transposon mutants. This study showed that recombinant CyaB produced cAMP in a temperature-dependent manner but was not evaluated directly in *B. burgdorferi.* Our attempts to measure cAMP production during cultivation of *B. burgdorferi* have been unsuccessful perhaps due to the low-level expression and production of *cyaB* under these conditions. Transposon mutants with insertions in *cyaB* (insertion ratios 0.02 and 0.93) did not demonstrate an infectivity phenotype by needle inoculation or tick transmission (Khajanchi et al., 2016). Infectivity was qualitatively evaluated by borrelial outgrowth from infected tissues following a 28-day infection in which all tissues were positive for the presence of the bacteria. Our findings observed *B. burgdorferi* in most tissues at the last time point with the exception of the ears that were not assessed in the prior work. We quantitated the borrelial load of the whole mouse and individual tissues by bioluminescent imaging and qPCR, respectively. We found that overall borrelial loads were reduced that were specifically lower in the skin flank and bladders during early infection, but remained low in the ears and the tibiotarsal joints. We have also investigated the involvement of SR0623 that was unknown at the time of previous studies. This indicates that *cyaB* may contribute to pathogenesis during earlier borrelial infection that can be overcome by compensatory but unknown genes, to be able to reach a fully disseminated infection. Our study further pursued the role of borrelial ACs by investigating the regulatory effect of *cyaB* on known borrelial virulence determinants. While this study focused on a few targets, it is likely that *cyaB* and cAMP have a broader impact on transcriptional and posttranscriptional regulation in *B. burgdorferi*.

Another important aspect of bacterial posttranscriptional regulation is the contribution by sRNAs. Over a thousand sRNAs were recently identified in *B. burgdorferi* (Popitsch et al., 2017), but few have been characterized. SR0623 is an intragenic sRNA that is encoded on the negative strand within the 3′ end of *cyaB* (*bb0723*) and overlaps with the hypothetical gene *bb0722* (Figure 1). SR0623 is predicted to be transcribed with *cyaB* and processed, which could result in a truncated *cyaB* transcript. Intragenic sRNAs in other bacteria regulate the genes they are encoded within; therefore, SR0623 may regulate *cyaB* and/or *bb0722*. In addition, intragenic RNAs have been co-immunoprecipitated with RNA-binding proteins and other mRNAs and sRNAs, indicating they may have multiple targets besides the genes they are encoded within (Bilusic et al., 2014; Melamed et al., 2016, 2020; Iosub et al., 2020). Northern blots were used to compare steady-state transcript levels between the different *cyaB* complement strains and to differentiate between full-length *cyaB* transcript, processed or degraded *cyaB* transcript, and SR0623. In the complement with truncated SR0623, there are lower steady-state levels of *cyaB* compared with the WT, indicating SR0623 and/or the 3′ end of the *cyaB* transcript is important for regulating *cyaB* steady-state transcript levels. Furthermore, in the *cyaB* full-length SR0623 complement, there are higher steady-state levels of *cyaB* and SR0623. Interestingly, both ACs and sRNAs function in posttranscriptional regulation. Finally, we also cannot rule out that our observed phenotype and challenges with complementation may be in part attributed to SR0623 regulation of *bb0722* or the intra-RNA SR0622 encoded within it.

The current study does not address the direct detection of cAMP from cultivated *B. burgdorferi*. Future studies will investigate the environmental conditions *in vitro* and *in vivo* that promote the production of cAMP, and determine if it correlates with the expression or production of *cyaB*. Herein, we narrowly focused on the regulation of known tick and mammalian virulence determinant, but it is likely that *cyaB* and cAMP have a broader posttranscriptional regulatory impact on *B. burgdorferi.* The various strategies used to complement *cyaB* and/or SR0623 resulted in restoration phenotypes under *in vitro* conditions, but unfortunately, we were not able to completely restore the WT phenotype during mouse infection. This could be due to complementation of *cyaB* between *bb0445* and *bb0446* rather than at its native locus because of the difficult gene arrangement with overlapping *bb0772* and SR0623 (Figure 1). Successful phenotypic complementation at this locus was initially seen by Li et al. (2007) and has been used by others in the field; however, we cannot rule out the possibility that complementation at this location may have altered the genomic landscape and affected complementation. In addition, the lack of *in vivo* complementation could be due to altered processing or stability of SR0623 and/or *cyaB* in the complement strains, which may impact the AC activity specifically induced under host-adapted conditions. Our study is not the first to have difficulty complementing a borrelial gene or sRNA and represents a broader challenge in the field of bacterial pathogenesis. Further study is required to distinguish the function of intergenic sRNA from the gene it is encoded within to fully understand the complex regulation of *B. burgdorferi*.

In this study, we identified the ability of *cyaB* to contribute to the regulation of mammalian virulence determinants and infectivity in mice. This phenotype is presumably due to the production of cAMP and its impact on posttranscriptional regulation in *B. burgdorferi.* It also shed light on the complexities and possible contribution of sRNA to borrelial regulation in which the distinct responses are observed under cultivation conditions and/or during infection. It has become clear that *B. burgdorferi* utilizes posttranscriptional regulation to support pathogenesis and to provide a dynamic means to adapt to the various milieus that Lyme spirochetes move between during its complex life cycle.

## Data Availability Statement

The raw data supporting the conclusions of this article will be made available by the authors, without undue reservation.

## Ethics Statement

Texas A&M University is accredited by the Association for Assessment and Accreditation of Laboratory Animal Care (AAALAC) indicating their commitment to responsible animal care and use. All animal experiments were performed in accordance with the Guide for the Care and Use of Laboratory Animals provided by the National Institutes of Health (NIH) and the Guidelines of the Approval for Animal Procedures provided by the Institutional Animal Care and Use Committee (IACUC) at Texas A&M University.

## Author Contributions

VA and JH were involved in the experimental design and data analysis and interpretation and wrote the manuscript. VA executed majority of the experiments. LF conducted the mammalian tissue culture experiments. ES generated the constructs and strains. AH and ML conducted the northern blots. ML was responsible for the consultation, experimental design, and data analysis and interpretation. NO’B and RM were responsible for the antibody design and generation. AO conducted the tick tissue experiments. All authors were involved in manuscript editing.

## Conflict of Interest

The authors declare that the research was conducted in the absence of any commercial or financial relationships that could be construed as a potential conflict of interest.

## Supplementary Material

Supplementary Figure 1 SR0623 wobble mutation sequence. Site directed mutagenesis of every third base pair of the sRNA SR0623 sequence is denoted by underlining. The *cyaB* ORF stop codon is indicated by an asterisk. The stop codon for the overlapping *bb0722* ORF is outlined by a box. The numbers indicate the distance from the *cyaB* ORF start codon.

Supplementary Figure 2 Glycerol does not alter *B. burgdorferi* virulence factor production. The *B. burgdorferi* strains 5A4-NP1 (WT) and JH522 (*cyaB*^–^) were grown in BSK-lite with or without 0.6% glycerol to mid-logarithmic phase at 32°C 1% CO_2_. Protein was harvested and resolved on a SDS-PAGE with each lane containing approximately 4 × 10^7^ *B. burgdorferi*. Immunoblotting was carried out using the anti-serum depicted. FlaB was used as a loading control. Representative of at least three independent replicates.

Supplementary Figure 3 Mammalian virulence factors production in bioluminescent *cyaB* mutant. *B. burgdorferi* ML23 strains were grown in BSK-glycerol to mid-log phase at 32°C 1% CO_2_. Protein was harvested and resolved on a SDS-PAGE with each lane containing approximately 4 × 10^7^ *B. burgdorferi*. Immunoblots were probed using the anti-serum depicted. FlaB was used as a loading control. Representative of at least three individual replicates. The following abbreviations are used to indicate strains: ML23 pBBE22*luc* (P), JH441 pBBE22*luc* (M), JH446 pBBE22*luc* (C).

Supplementary Table 1 Primers used in this study.

## References

[B1] Adams P. P. Flores Avile C. Popitsch N. Bilusic I. Schroeder R. Lybecker M. (2017). In vivo expression technology and 5′ end mapping of the borrelia burgdorferi transcriptome identify novel RNAs expressed during mammalian infection. *Nucleic Acids Res.* 45 775–792. 10.1093/nar/gkw1180 27913725 PMC5314773

[B2] Aline Dias da P. Nathalia Marins de A. Gabriel Guarany de A. Robson Francisco de S. Cristiane Rodrigues G. (2020). The world of cyclic dinucleotides in bacterial behavior. *Molecules* 25:2462. 10.3390/molecules25102462 32466317 PMC7288161

[B3] Babitzke P. Lai Y. J. Renda A. J. Romeo T. (2019). Posttranscription initiation control of gene expression mediated by bacterial RNA-binding proteins. *Ann. Rev. Microbiol.* 73 43–67. 10.1146/annurev-micro-020518-115907 31100987 PMC9404307

[B4] Barbour A. G. (1984). Isolation and cultivation of lyme disease spirochetes. *Yale J. Biol. Med.* 57 521–525. 6393604 PMC2589996

[B5] Bilusic I. Popitsch N. Rescheneder P. Schroeder R. Lybecker M. (2014). Revisiting the coding potential of the E. Coli genome through Hfq Co-immunoprecipitation. *RNA Biol.* 11 641–654. 10.4161/rna.29299 24922322 PMC4152368

[B6] Blevins J. S. Revel A. T. Smith A. H. Bachlani G. N. Norgard M. V. (2007). Adaptation of a luciferase gene reporter and lac expression system to borrelia burgdorferi. *Appl. Environ. Microbiol.* 73 1501–1513. 10.1128/AEM.02454-06 17220265 PMC1828772

[B7] Blevins J. S. Xu H. He M. Norgard M. V. Reitzer L. Yang X. F. (2009). Rrp2, a Σ54-dependent transcriptional activator of borrelia burgdorferi, activates RpoS in an enhancer-independent manner. *J. Bacteriol.* 191 2902–2905. 10.1128/JB.01721-08 19201806 PMC2668385

[B8] Bontemps-Gallo S. Lawrence K. Gherardini F. C. (2016). Two different virulence-related regulatory pathways in borrelia burgdorferi are directly affected by osmotic fluxes in the blood meal of feeding ixodes ticks. *PLoS Pathog.* 12:e1005791. 10.1371/journal.ppat.1005791 27525653 PMC4985143

[B9] Botsford J. L. Harman J. G. (1992). Cyclic AMP in prokaryotes. *Microbiol. Mol. Biol. Rev.* 56 100–122. 10.1128/mr.56.1.100-122.1992 PMC372856 1315922

[B10] Boylan J. A. Hummel C. S. Benoit S. Garcia-Lara J. Treglown-Downey J. Crane E. J. (2006). Borrelia burgdorferi Bb0728 encodes a coenzyme a disulphide reductase whose function suggests a role in intracellular redox and the oxidative stress response. *Mol. Microbiol.* 59 475–486. 10.1111/j.1365-2958.2005.04963.x 16390443

[B11] Boylan J. A. Lawrence K. A. Downey J. S. Gherardini F. C. (2008). Borrelia burgdorferi membranes are the primary targets of reactive oxygen species. *Mol. Microbiol.* 68 786–799. 10.1111/j.1365-2958.2008.06204.x 18373524 PMC2327290

[B12] Boylan J. A. Posey J. E. Gherardini F. C. (2003). Borrelia oxidative stress response regulator, BosR: a distinctive Zn-dependent transcriptional activator. *Proc. Natl. Acad. Sci. U S A.* 100 11684–11689. 10.1073/pnas.2032956100 12975527 PMC208818

[B13] Caimano M. J. Dunham-Ems S. Allard A. M. Cassera M. B. Kenedy M. Radolf J. D. (2015). C-Di-GMP modulates gene expression in lyme disease spirochetes at the tick-mammal interface to promote spirochete survival during the blood meal and tick-to-mammal transmission. *Infect. Immun.* 83 3043–3060. 10.1128/IAI.00315-15 25987708 PMC4496621

[B14] Caimano M. J. Eggers C. H. Gonzalez C. A. Radolf J. D. (2005). Alternate sigma factor RpoS Is required for the in vivo-specific repression of borrelia burgdorferi plasmid Lp54-borne OspA and Lp6.6 genes. *J. Bacteriol.* 187 7845–7852. 10.1128/JB.187.22.7845-7852.2005 16267308 PMC1280317

[B15] Caimano M. J. Eggers C. H. Hazlett K. R. Radolf J. D. (2004). RpoS is not central to the general stress response in borrelia burgdorferi but does control expression of one or more essential virulence determinants. *Infect. Immun.* 72 6433–6445. 15501774 10.1128/IAI.72.11.6433-6445.2004 PMC523033

[B16] Caimano M. J. Groshong A. M. Belperron A. Mao J. Hawley K. L. Luthra A. (2019). The RpoS gatekeeper in borrelia burgdorferi: an invariant regulatory scheme that promotes spirochete persistence in reservoir hosts and niche diversity. *Front. Microbiol.* 10:1923. 10.3389/fmicb.2019.01923 31507550 PMC6719511

[B17] Caimano M. J. Iyer R. Eggers C. H. Gonzalez C. Morton E. A. Gilbert M. A. (2007). Analysis of the RpoS regulon in borrelia burgdorferi in response to mammalian host signals provides insight into RpoS function during the enzootic cycle. *Mol. Microbiol.* 65 1193–1217. 10.1111/j.1365-2958.2007.05860.x 17645733 PMC2967192

[B18] Cann M. J. Hammer A. Zhou J. Kanacher T. (2003). A defined subset of adenylyl cyclases is regulated by bicarbonate Ion. *J. Biol. Chem.* 278 35033–35038. 10.1074/jbc.M303025200 12829712

[B19] Carroll J. A. Cordova R. M. Garon C. F. (2000). Identification of 11 PH-regulated genes in borrelia burgdorferi localizing to linear plasmids. *Infect. Immun.* 68 6677–6684. 10.1128/iai.68.12.6677-6684.2000 11083781 PMC97766

[B20] Carroll J. A. Garon C. F. Schwan T. G. (1999). Effects of environmental PH on membrane proteins in borrelia burgdorferi. *Infect. Immun.* 67 3181–3187. 10377088 10.1128/iai.67.7.3181-3187.1999 PMC116493

[B21] Casjens S. Palmer N. van Vugt R. Huang W. M. Stevenson B. Rosa P. (2000). A bacterial genome in flux: the twelve linear and nine circular extrachromosomal DNAs in an infectious isolate of the lyme disease spirochete borrelia burgdorferi. *Mol. Microbiol.* 35 490–516. 10.1046/j.1365-2958.2000.01698.x 10672174

[B22] Cugini C. Medrano M. Schwan T. G. Coburn J. (2003). Regulation of expression of the borrelia burgdorferi beta(3)-chain integrin ligand, P66, in ticks and in culture. *Infect. Immun.* 71 1001–1007. 10.1128/iai.71.2.1001-1007.2003 12540584 PMC145366

[B23] Curtiss R. Kelly S. M. (1987). *Salmonella* typhimurium deletion mutants lacking adenylate cyclase and cyclic AMP receptor protein are avirulent and immunogenic. *Infect. Immun.* 55 3035–3043. 10.1128/IAI.55.12.3035-3043.1987 3316029 PMC260025

[B24] Dong Q. Yan X. Zheng M. Yang Z. (2013). Comparison of two type IV hyperthermophilic adenylyl cyclases characterizations from the archaeon *Pyrococcus Furiosus*. *J. Mol. Catalysis B Enzymatic* 88 7–13. 10.1016/j.molcatb.2012.10.017

[B25] Drecktrah D. Hall L. S. Rescheneder P. Lybecker M. Samuels D. S. (2018). The stringent response-regulated SRNA transcriptome of borrelia burgdorferi. *Front. Cell. Infect. Microbiol.* 8:231. 10.3389/fcimb.2018.00231 30027068 PMC6041397

[B26] El Mouali Y. Gaviria-Cantin T. Sánchez-Romero M. A. Gibert M. Westermann A. J. Vogel J. (2018). CRP-CAMP mediates silencing of *Salmonella* virulence at the post-transcriptional level. *PLoS Genet.* 14:e1007401. 10.1371/journal.pgen.1007401 29879120 PMC5991649

[B27] Elias A. F. Bono J. L. Kupko J. J. III Stewart P. E. Krum J. G. Rosa P. A. (2003). New antibiotic resistance cassettes suitable for genetic studies in borrelia burgdorferi. *J. Mol. Microbiol. Biotechnol.* 6 29–40. 10.1159/000073406 14593251

[B28] Franchini A. G. Ihssen J. Egli T. (2015). Effect of global regulators RpoS and cyclic-AMP/CRP on the catabolome and transcriptome of *Escherichia Coli* K12 during carbon- and energy-limited growth. *PloS One* 10:e0133793. 10.1371/journal.pone.0133793 26204448 PMC4512719

[B29] Freedman J. C. Rogers E. A. Kostick J. L. Zhang H. Iyer R. Schwartz I. (2010). Identification and molecular characterization of a cyclic-Di-GMP effector protein, PlzA (BB0733): additional evidence for the existence of a functional cyclic-Di-GMP regulatory network in the lyme disease spirochete, borrelia burgdorferi. *FEMS Immunol. Med. Microbiol.* 58 285–294. 10.1111/j.1574-695X.2009.00635.x 20030712 PMC2868932

[B30] Gallagher D. T. Smith N. N. Kim S. K. Heroux A. Robinson H. Reddy P. T. (2006). Structure of the class IV adenylyl cyclase reveals a novel fold. *J. Mol. Biol.* 362 114–122. 10.1016/j.jmb.2006.07.008 16905149

[B31] Gottesman S. Storz G. (2011). Bacterial small RNA regulators: versatile roles and rapidly evolving variations. *Cold Spring Harb. Perspect. Biol.* 3:a003798. 10.1101/cshperspect.a003798 20980440 PMC3225950

[B32] Gstrein-Reider E. Schweiger M. (1982). Regulation of adenylate cyclase in E. Coli. *EMBO J.* 1 333–337. 6325159 10.1002/j.1460-2075.1982.tb01170.x PMC553045

[B33] Guo B. P. Brown E. L. Dorward D. W. Rosenberg L. C. Hook M. (1998). Decorin-binding adhesins from borrelia burgdorferi. *Mol. Microbiol.* 30 711–723. 10094620 10.1046/j.1365-2958.1998.01103.x

[B34] He M. Boardman B. K. Yan D. Yang X. F. (2007). Regulation of expression of the fibronectin-binding protein BBK32 in borrelia burgdorferi. *J. Bacteriol.* 189 8377–8380. 10.1128/JB.01199-07 17873053 PMC2168679

[B35] He M. Ouyang Z. Troxell B. Xu H. Moh A. Piesman J. (2011). Cyclic Di-GMP is essential for the survival of the lyme disease spirochete in ticks. *PLoS Pathog.* 7:e1002133. 10.1371/journal.ppat.1002133 21738477 PMC3128128

[B36] He M. Zhang J. J. Ye M. Lou Y. Yang X. F. (2014). Cyclic Di-GMP receptor PlzA controls virulence gene expression through RpoS in borrelia burgdorferi. *Infect. Immun.* 82 445–452. 10.1128/IAI.01238-13 24218478 PMC3911845

[B37] Hoffmaster A. R. Koehler T. M. (1997). The anthrax toxin activator gene AtxA is associated with CO2-enhanced non-toxin gene expression in *Bacillus anthracis*. *Infect. Immun.* 65 3091–3099. 10.1128/IAI.65.8.3091-3099.1997 9234759 PMC175436

[B38] Hu L. T. (2016). Lyme disease. *Ann. Intern. Med.* 165:677. 10.7326/L16-0409 27802469

[B39] Hübner A. Yang X. Nolen D. M. Popova T. G. Cabello F. C. Norgard M. V. (2001). Expression of borrelia burgdorferi OspC and DbpA is controlled by a RpoN-RpoS regulatory pathway. *Proc. NatL. Acad. Sci. USA.* 98 12724–12729. 10.1073/pnas.231442498 11675503 PMC60121

[B40] Hyde J. A. Shaw D. K. Smith Iii R Trzeciakowski J. P. Skare J. T. (2009). The BosR regulatory protein of borrelia burgdorferi interfaces with the RpoS regulatory pathway and modulates both the oxidative stress response and pathogenic properties of the lyme disease spirochete. *Mol. Microbiol.* 74 1344–1355. 10.1111/j.1365-2958.2009.06951.x 19906179 PMC2805275

[B41] Hyde J. A. Shaw D. K. Smith R. III Trzeciakowski J. P. Skare J. T. (2010). Characterization of a conditional BosR mutant in borrelia burgdorferi. *Infect. Immun.* 78 265–274. 10.1128/IAI.01018-09 19858309 PMC2798208

[B42] Hyde J. A. Skare J. T. (2018). Detection of bioluminescent borrelia burgdorferi from in vitro cultivation and during murine infection. *Methods Mol. Biol.* 1690 241–257. 10.1007/978-1-4939-7383-5_18 29032549 PMC8786108

[B43] Hyde J. A. Trzeciakowski J. P. Skare J. T. (2007). Borrelia burgdorferi alters its gene expression and antigenic profile in response to CO2 levels. *J. Bacteriol.* 189 437–445. 10.1128/JB.01109-06 17098904 PMC1797391

[B44] Hyde J. A. Weening E. H. Chang M. Trzeciakowski J. P. Höök M. Cirillo J. D. (2011a). Bioluminescent imaging of borrelia burgdorferi in vivo demonstrates that the fibronectin-binding protein BBK32 is required for optimal infectivity. *Mol. Microbiol.* 82 99–113. 10.1111/j.1365-2958.2011.07801.x 21854463 PMC3183165

[B45] Hyde J. A. Weening E. H. Skare J. T. (2011b). Genetic transformation of borrelia burgdorferi. *Curr. Protoc. Microbiol.* 20 1–12. 10.1002/9780471729259.mc12c04s20 21400675 PMC3561735

[B46] Iosub I. A. van Nues R. W. McKellar S. W. Nieken K. J. Marchioretto M. Sy B. (2020). Hfq clash uncovers SRNA-target interaction networks linked to nutrient availability adaptation. *ELife* 9:e54655. 10.7554/eLife.54655 32356726 PMC7213987

[B47] Izac J. R. Camire A. C. Earnhart C. G. Embers M. E. Funk R. A. Breitschwerdt E. B. (2019). Analysis of the antigenic determinants of the OspC protein of the lyme disease spirochetes: evidence that the C10 motif is not immunodominant or required to elicit bactericidal antibody responses. *Vaccine* 37 2401–2407. 10.1016/j.vaccine.2019.02.007 30922701 PMC6453540

[B48] Kamenetsky M. Middelhaufe S. Bank E. M. Levin L. R. Buck J. Steegborn C. (2006). Molecular details of CAMP generation in mammalian cells: a tale of two systems. *J. Mol. Biol.* 362 623–639. 10.1016/j.jmb.2006.07.045 16934836 PMC3662476

[B49] Kawabata H. Norris S. J. Watanabe H. (2004). BBE02 disruption mutants of borrelia burgdorferi B31 have a highly transformable, infectious phenotype. *Infect. Immun.* 72 7147–7154. 10.1128/IAI.72.12.7147-7154.2004 15557639 PMC529111

[B50] Khajanchi B. K. Odeh E. Gao L. Jacobs M. B. Philipp M. T. Lin T. (2016). Phosphoenolpyruvate phosphotransferase system components modulate gene transcription and virulence of borrelia burgdorferi. *Infect. Immun.* 84 754–764. 10.1128/IAI.00917-15 26712207 PMC4771366

[B51] Konkel M. E. Tilly K. (2000). Temperature-regulated expression of bacterial virulence genes. *Microb. Infect.* 2 157–166. 10.1016/s1286-4579(00)00272-0 10742688

[B52] Kostick J. L. Szkotnicki L. T. Rogers E. A. Bocci P. Raffaelli N. Marconi R. T. (2011). The diguanylate cyclase, Rrp1, regulates critical steps in the enzootic cycle of the lyme disease spirochetes. *Mol. Microbiol.* 81 219–231. 10.1111/j.1365-2958.2011.07687.x 21542866 PMC3124615

[B53] Kostick-Dunn J. L. Izac J. R. Freedman J. C. Szkotnicki L. T. Oliver L. D. Jr. Marconi R. T. (2018). The borrelia burgdorferi C-Di-GMP binding receptors, PlzA and PlzB, are functionally distinct. *Front. Cell. Infect. Microbiol.* 8:213. 10.3389/fcimb.2018.00213 30050868 PMC6050380

[B54] Labandeira-Rey M. Skare J. T. (2001). Decreased infectivity in borrelia burgdorferi strain B31 is associated with loss of linear plasmid 25 or 28-1. *Infect. Immun.* 69 446–455. 10.1128/IAI.69.1.446-455.2001 11119536 PMC97902

[B55] Lawrenz M. B. Kawabata H. Purser J. E. Norris S. J. (2002). Decreased electroporation efficiency in borrelia burgdorferi containing linear plasmids Lp25 and Lp56: impact on transformation of infectious B burgdorferi. *Infect. Immun.* 70 4798–4804. 10.1128/iai.70.9.4798-4804.2002 12183522 PMC128261

[B56] Li X. Pal U. Ramamoorthi N. Liu X. Desrosiers D. C. Eggers C. H. (2007). The lyme disease agent borrelia burgdorferi requires BB0690, a Dps homologue, to persist within ticks. *Mol. Microbiol.* 63 694–710. 10.1111/j.1365-2958.2006.05550.x 17181780

[B57] Liang W. Pascual-Montano A. Silva A. J. Benitez J. A. (2007). The cyclic AMP receptor protein modulates quorum sensing, motility and multiple genes that affect intestinal colonization in *Vibrio Cholerae*. *Microbiology* 153 2964–2975. 10.1099/mic.0.2007/006668-0 17768239

[B58] Liu C. Sun D. Zhu J. Liu J. Liu W. (2020). The regulation of bacterial biofilm formation by CAMP-CRP: a mini-review. *Front. Microbiol.* 11:802. 10.3389/fmicb.2020.00802 32528421 PMC7247823

[B59] Livak K. J. Schmittgen T. D. (2001). Analysis of relative gene expression data using real-time quantitative PCR and the 2(-delta delta C(T)) method. *Methods* 25 402–408. 10.1006/meth.2001.1262 11846609

[B60] Livengood J. A. Schmit V. L. Gilmore R. D. Jr. (2008). Global transcriptome analysis of borrelia burgdorferi during association with human neuroglial cells. *Infect. Immun.* 76 298–307. 10.1128/IAI.00866-07 17984208 PMC2223675

[B61] Lybecker M. Henderson K. C. (2018). Borrelia burgdorferi transcriptome analysis by RNA-sequencing. *Methods Mol. Biol.* 1690 127–136. 29032542 10.1007/978-1-4939-7383-5_11

[B62] Lybecker M. Zimmermann B. Bilusic I. Tukhtubaeva N. Schroeder R. (2014). The double-stranded transcriptome of *Escherichia Coli*. *Proc. Natl. Acad. Sci. USA.* 111 3134–3139. 10.1073/pnas.1315974111 24453212 PMC3939876

[B63] Mallory K. L. Miller D. P. Oliver L. D. Jr. Freedman J. C. Kostick-Dunn J. L. Carlyon J. A. (2016). Cyclic-Di-GMP binding induces structural rearrangements in the PlzA and PlzC proteins of the lyme disease and relapsing fever spirochetes: a possible switch mechanism for c-Di-GMP-mediated effector functions. *Pathog. Dis.* 74:ftw105. 10.1093/femspd/ftw105 27852620

[B64] Maruskova M. Esteve-Gassent M. D. Sexton V. L. Seshu J. (2008). Role of the BBA64 locus of borrelia burgdorferi in early stages of infectivity in a murine model of lyme disease. *Infect. Immun.* 76 391–402. 10.1128/IAI.01118-07 17984202 PMC2223643

[B65] McDonough K. A. Rodriguez A. (2011). The myriad roles of cyclic AMP in microbial pathogens: from signal to sword. *Nat. Rev. Microbiol.* 10:27. 10.1038/nrmicro2688 22080930 PMC3785115

[B66] McKnight G. S. (1991). Cyclic AMP second messenger systems. *Curr. Opin. Cell. Biol.* 3 213–217. 10.1016/0955-0674(91)90141-K 1652989

[B67] Melamed S. Adams P. P. Zhang A. Zhang H. Storz G. (2020). RNA-RNA interactomes of ProQ and Hfq reveal overlapping and competing roles. *Mol. Cell.* 77 411–425. 10.1016/j.molcel.2019.10.022 31761494 PMC6980735

[B68] Melamed S. Peer A. Faigenbaum-Romm R. Gatt Y. E. Reiss N. Bar A. (2016). Global mapping of small RNA-target interactions in bacteria. *Mol. Cell.* 63 884–897. 10.1016/j.molcel.2016.07.026 27588604 PMC5145812

[B69] Miller C. L. Karna S. L. Seshu J. (2013). Borrelia host adaptation regulator (BadR) regulates RpoS to modulate host adaptation and virulence factors in borrelia burgdorferi. *Mol. Microbiol.* 88 105–124. 10.1111/mmi.12171 23387366 PMC4828661

[B70] Miller D. P. Oliver L. D. Jr. Tegels B. K. Reed L. A. O’Bier N. S. Kurniyati K. (2016). The treponema denticola FhbB protein is a dominant early antigen that elicits FhbB variant-specific antibodies that block factor H binding and cleavage by dentilisin. *Infect. Immun.* 84 2051–2058. 10.1128/IAI.01542-15 27113359 PMC4936362

[B71] Molina-Quiroz R. C. Silva-Valenzuela C. Brewster J. Castro-Nallar E. Levy S. B. Camilli A. (2018). Cyclic AMP regulates bacterial persistence through repression of the oxidative stress response and SOS-dependent DNA repair in uropathogenic *Escherichia Coli*. *MBio* 9 e2117–e2144. 10.1128/mBio.02144-17 29317513 PMC5760743

[B72] Novak E. A. Sultan S. Z. Motaleb M. A. (2014). The cyclic-di-gmp signaling pathway in the lyme disease spirochete, borrelia burgdorferi. *Front. Cell. Infect. Microbiol.* 4:56. 10.3389/fcimb.2014.00056 24822172 PMC4013479

[B73] Oliver J. D. Chávez A. S. Felsheim R. F. Kurtti T. J. Munderloh U. G. (2015). An ixodes scapularis cell line with a predominantly neuron-like phenotype. *Exper. Appl. Acarol.* 66 427–442. 10.1007/s10493-015-9908-1 25894426 PMC4449809

[B74] Oliver J. H. Chandler F. W. Luttrell M. P. James A. M. Stallknecht D. E. McGuire B. S. (1993). Isolation and transmission of the lyme disease spirochete from the southeastern united states. *Proc. Natl. Acad. Sci. USA.* 90 7371–7375. 8346258 10.1073/pnas.90.15.7371 PMC47139

[B75] Ouyang Z. Blevins J. S. Norgard M. V. (2008). Transcriptional interplay among the regulators Rrp2, RpoN and RpoS in borrelia burgdorferi. *Microbiology* 154 2641–2658. 10.1099/mic.0.2008/019992-0 18757798

[B76] Ouyang Z. Deka R. K. Norgard M. V. (2011). BosR (BB0647) controls the RpoN-RpoS regulatory pathway and virulence expression in borrelia burgdorferi by a novel DNA-binding mechanism. *PLoS Pathog.* 7:e1001272. 10.1371/journal.ppat.1001272 21347346 PMC3037356

[B77] Ouyang Z. Kumar M. Kariu T. Haq S. Goldberg M. Pal U. (2009). BosR (BB0647) governs virulence expression in borrelia burgdorferi. *Mol. Microbiol.* 74 1331–1343. 10.1111/j.1365-2958.2009.06945.x 19889086 PMC2831293

[B78] Papenfort K. Vogel J. (2010). Regulatory RNA in bacterial pathogens. *Cell Host. Microbe* 8 116–127. 10.1016/j.chom.2010.06.008 20638647

[B79] Peterkofsky A. Gazdar C. (1974). Glucose inhibition of adenylate cyclase in intact cells of *Escherichia Coli* B. *Proc. Natl. Acad. Sci. USA.* 71 2324–2328. 10.1073/pnas.71.6.2324 4366761 PMC388445

[B80] Popitsch N. Bilusic I. Rescheneder P. Schroeder R. Lybecker M. (2017). Temperature-dependent SRNA transcriptome of the lyme disease spirochete. *BMC Genom.* 18:28. 10.1186/s12864-016-3398-3 28056764 PMC5216591

[B81] Radolf J. D. Caimano M. J. Stevenson B. Hu L. T. (2012). Of ticks, mice and men: understanding the dual-host lifestyle of lyme disease spirochaetes. *Nat. Rev. Microbiol.* 10 87–99. 10.1038/nrmicro2714 22230951 PMC3313462

[B82] Ramsey M. E. Hyde J. A. Medina-Perez D. N. Lin T. Gao L. Lundt M. E. (2017). A high-throughput genetic screen identifies previously uncharacterized borrelia burgdorferi genes important for resistance against reactive oxygen and nitrogen species. *PLoS Pathog.* 13:e1006225. 10.1371/journal.ppat.1006225 28212410 PMC5333916

[B83] Rebollo-Ramirez S. Larrouy-Maumus G. (2019). NaCl triggers the CRP-dependent increase of CAMP in *Mycobacterium Tuberculosis*. *Tuberculosis* 116 8–16. 10.1016/j.tube.2019.03.009 31153521

[B84] Rogers E. A. Terekhova D. Zhang H. M. Hovis K. M. Schwartz I. Marconi R. T. (2009). Rrp1, a cyclic-Di-GMP-producing response regulator, is an important regulator of borrelia burgdorferi core cellular functions. *Mol. Microbiol.* 71 1551–1573. 10.1111/j.1365-2958.2009.06621.x 19210621 PMC2843504

[B85] Rosenberg R. Lindsey N. P. Fischer M. Gregory C. J. Hinckley A. F. Mead P. S. (2018). Vital signs: trends in reported vectorborne disease cases — united states and territories, 2004–2016. *MMWR Morb. Mortal. Wkly Rep.* 67 496–501. 10.15585/mmwr.mm6717e1 29723166 PMC5933869

[B86] Samuels D. S. Mach K. E. Garon C. F. (1994). Genetic transformation of the lyme disease agent borrelia burgdorferi with coumarin-resistant GyrB. *J. Bacteriol.* 176 6045–6049. 10.1128/jb.176.19.6045-6049.1994 7928965 PMC196823

[B87] Samuels D. S. Samuels L. R. N. (2016). Gene regulation during the enzootic cycle of the lyme disease spirochete. *For. Immunopathol. Dis. Therap.* 7 205–212. 10.1615/ForumImmunDisTher.2017019469 29876141 PMC5985821

[B88] Saputra E. P. Trzeciakowski J. P. Hyde J. A. (2020). Borrelia burgdorferi spatiotemporal regulation of transcriptional regulator BosR and decorin binding protein during murine infection. *Sci. Rep.* 10:12534. 10.1038/s41598-020-69212-7 32719448 PMC7385660

[B89] Savage C. R. Arnold W. K. Gjevre-Nail A. Koestler B. J. Bruger E. L. Barker J. R. (2015). Intracellular concentrations of borrelia burgdorferi cyclic Di-AMP are not changed by altered expression of the CdaA synthase. *PloS One* 10:e0125440. 10.1371/journal.pone.0125440 25906393 PMC4408052

[B90] Schmit V. L. Patton T. G. Gilmore R. D. Jr. (2011). Analysis of borrelia burgdorferi surface proteins as determinants in establishing host cell interactions. *Front. Microbiol.* 2:141. 10.3389/fmicb.2011.00141 21747816 PMC3129520

[B91] Seshu J. Boylan J. A. Gherardini F. C. Skare J. T. (2004a). Dissolved oxygen levels alter gene expression and antigen profiles in borrelia burgdorferi. *Infect. Immun.* 72 1580–1586. 14977964 10.1128/IAI.72.3.1580-1586.2004 PMC356058

[B92] Seshu J. Boylan J. A. Hyde J. A. Swingle K. L. Gherardini F. C. Skare J. T. (2004b). A conservative amino acid change alters the function of BosR, the redox regulator of borrelia burgdorferi. *Mol. Microbiol.* 54 1352–1363. 10.1111/j.1365-2958.2004.04352.x 15554974

[B93] Sismeiro O. Trotot P. Biville F. Vivares C. Danchin A. (1998). Aeromonas hydrophila adenylyl cyclase 2: a new class of adenylyl cyclases with thermophilic properties and sequence similarities to proteins from hyperthermophilic archaebacteria. *J. Bacteriol.* 180 3339–3344. 10.1128/JB.180.13.3339-3344.1998 9642185 PMC107287

[B94] Smith A. H. Blevins J. S. Bachlani G. N. Yang X. F. Norgard M. V. (2007). Evidence that RpoS (SigmaS) in borrelia burgdorferi is controlled directly by RpoN (sigma54/sigman). *J. Bacteriol.* 189 2139–2144. 10.1128/JB.01653-06 17158681 PMC1855718

[B95] Smith N. Kim S. K. Reddy P. T. Gallagher D. T. (2006). Crystallization of the class IV adenylyl cyclase from yersinia pestis. *Acta Crystallogr. Sect. F Struct. Biol. Cryst. Commun.* 62 200–204. 10.1107/S1744309106002855 16511301 PMC2197185

[B96] Smith R. S. Wolfgang M. C. Lory S. (2004). An adenylate cyclase-controlled signaling network regulates *Pseudomonas Aeruginosa* virulence in a mouse model of acute pneumonia. *Infect. Immun.* 72 1677–1684. 10.1128/iai.72.3.1677-1684.2004 14977975 PMC356001

[B97] Stanek G. Strle F. (2018). Lyme borreliosis–from tick bite to diagnosis and treatment. *FEMS Microbiol. Rev.* 42 233–258. 10.1093/femsre/fux047 29893904

[B98] Steere A. C. Strle F. Wormser G. P. Hu L. T. Branda J. A. Hovius J. W. (2016). Lyme borreliosis. *Nat. Rev. Dis. Primers* 2:16090. 10.1038/nrdp.2016.90 27976670 PMC5539539

[B99] Stevenson B. Schwan T. G. Rosa P. A. (1995). Temperature-related differential expression of antigens in the lyme disease spirochete, borrelia burgdorferi. *Infect. Immun.* 63 4535–4539. 10.1128/IAI.63.11.4535-4539.1995 7591099 PMC173648

[B100] Sultan S. Z. Pitzer J. E. Boquoi T. Hobbs G. Miller M. R. Motaleb M. A. (2011). Analysis of the HD-GYP domain cyclic dimeric GMP phosphodiesterase reveals a role in motility and the enzootic life cycle of borrelia burgdorferi. *Infect. Immun.* 79 3273–3283. 10.1128/IAI.05153-11 21670168 PMC3147568

[B101] Sultan S. Z. Pitzer J. E. Miller M. R. Motaleb M. A. (2010). Analysis of a borrelia burgdorferi phosphodiesterase demonstrates a role for cyclic-Di-guanosine monophosphate in motility and virulence. *Mol. Microbiol.* 77 128–142. 10.1111/j.1365-2958.2010.07191.x 20444101 PMC2907449

[B102] Tokarz R. Anderton J. M. Katona L. I. Benach J. L. (2004). Combined effects of blood and temperature shift on borrelia burgdorferi gene expression as determined by whole genome DNA array. *Infect. Immun.* 72 5419–5432. 10.1128/IAI.72.9.5419-5432.2004 15322040 PMC517457

[B103] von Lackum K. Stevenson B. (2005). Carbohydrate utilization by the lyme borreliosis spirochete, borrelia burgdorferi. *FEMS Microbiol. Lett.* 243 173–179. 10.1016/j.femsle.2004.12.002 15668016

[B104] Wu J. Weening E. H. Faske J. B. Höök M. Skare J. T. (2011). Invasion of eukaryotic cells by borrelia burgdorferi requires B 1 integrins and src kinase activity. *Infect. Immun.* 79 1338–1348. 10.1128/IAI.01188-10 21173306 PMC3067508

[B105] Yang X. Goldberg M. S. Popova T. G. Schoeler G. B. Wikel S. K. Hagman K. E. (2000). Interdependence of environmental factors influencing reciprocal patterns of gene expression in virulent borrelia burgdorferi. *Mol. Microbiol.* 37 1470–1479. 10.1046/j.1365-2958.2000.02104.x 10998177

[B106] Yang X. F. Alani S. M. Norgard M. V. (2003). The response regulator Rrp2 is essential for the expression of major membrane lipoproteins in borrelia burgdorferi. *Proc. Natl. Acad. Sci.* 100 11001–11006. 10.1073/pnas.1834315100 12949258 PMC196916

[B107] Yang X. F. Lybecker M. C. Pal U. Alani S. M. Blevins J. Revel A. T. (2005). Analysis of the OspC regulatory element controlled by the RpoN-RpoS regulatory pathway in borrelia burgdorferi. *J. Bacteriol.* 187 4822– 4829. 15995197 10.1128/JB.187.14.4822-4829.2005 PMC1169512

[B108] Ye M. Zhang J. J. Fang X. Lawlis G. B. Troxell B. Zhou Y. (2014). DhhP, a cyclic Di-AMP phosphodiesterase of borrelia burgdorferi, is essential for cell growth and virulence. *Infect. Immun.* 82 1840–1849. 10.1128/IAI.00030-14 24566626 PMC3993442

[B109] Yin W. Cai X. Ma H. Zhu L. Zhang Y. Chou S. H. (2020). A decade of research on the second messenger C-Di-AMP. *FEMS Microbiol. Rev.* 44 701–724. 10.1093/femsre/fuaa019 32472931 PMC7850090

[B110] Zhang J. J. Chen T. Yang Y. Du J. Li H. Troxell B. (2018). Positive and negative regulation of glycerol utilization by the C-Di-GMP binding protein PlzA in borrelia burgdorferi. *J. Bacteriol.* 200 e218–e243. 10.1128/JB.00243-18 30181123 PMC6199477

