# Supplementary figures and images for: The Borrelia burgdorferi Adenylate Cyclase, CyaB, Is Important for Virulence Factor Production and Mammalian Infection
Source: Front Microbiol. 2021 May 25;12:676192. doi: 10.3389/fmicb.2021.676192 (PMC8186283; doi:10.3389/fmicb.2021.676192)

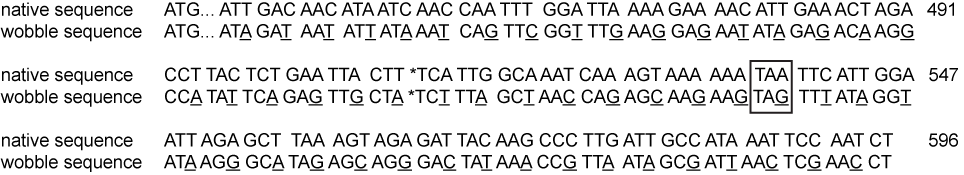

Supplement: Supplementary Figure 1 — SR0623 wobble mutation sequence. Site directed mutagenesis of every third base pair of the sRNA SR0623 sequence is denoted by underlining. The cyaB ORF stop codon is indicated by an asterisk. The stop codon for the overlapping bb0722 ORF is outlined by a box. The numbers indicate the distance from the cyaB ORF start codon. [file Image_1.TIF]

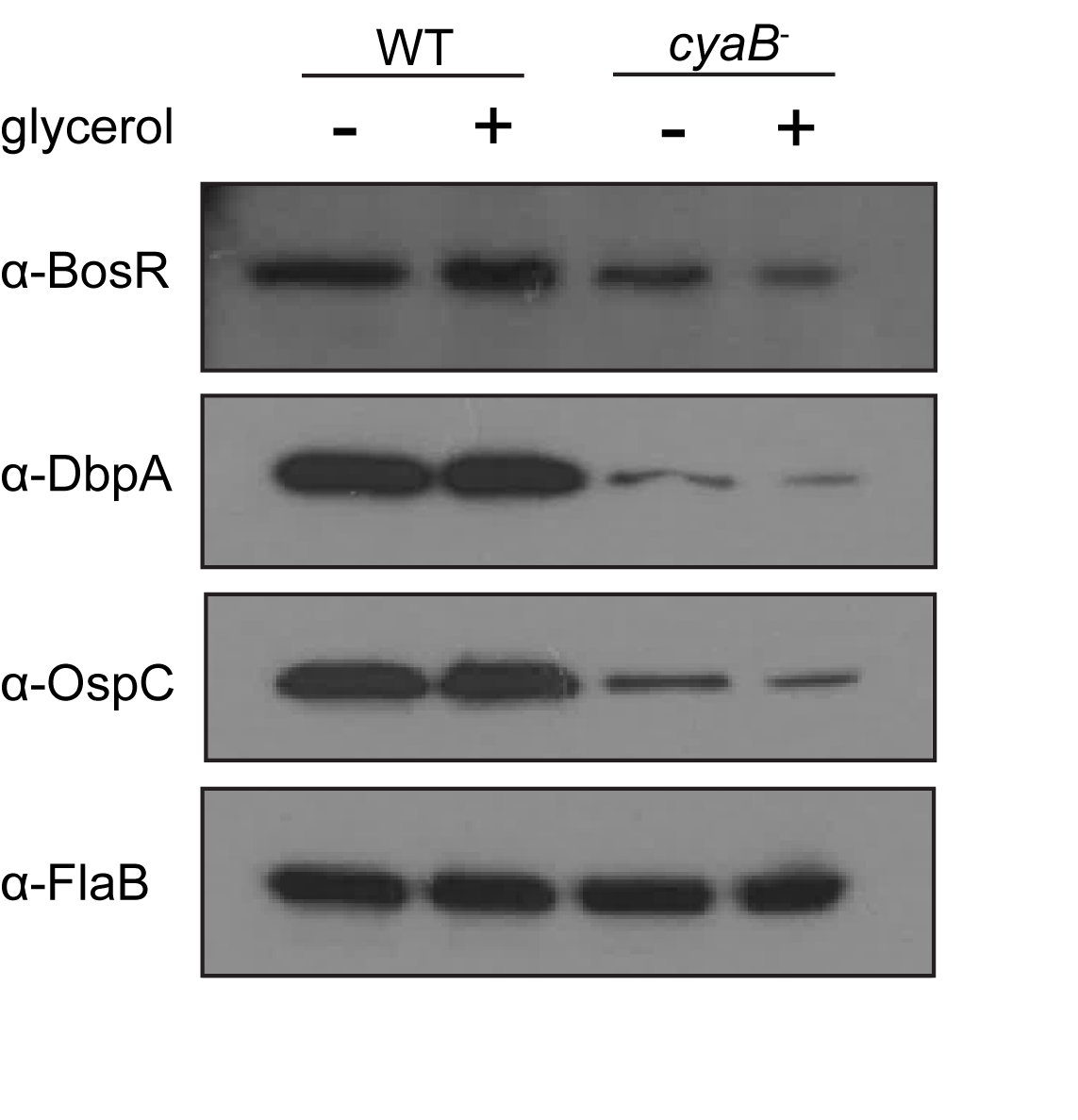

Supplement: Supplementary Figure 2 — Glycerol does not alter B. burgdorferi virulence factor production. The B. burgdorferi strains 5A4-NP1 (WT) and JH522 (cyaB–) were grown in BSK-lite with or without 0.6% glycerol to mid-logarithmic phase at 32°C 1% CO2. Protein was harvested and resolved on a SDS-PAGE with each lane containing approximately 4 × 10^7^ B. burgdorferi. Immunoblotting was carried out using the anti-serum depicted. FlaB was used as a loading control. Representative of at least three independent replicates. [file Image_2.TIF]

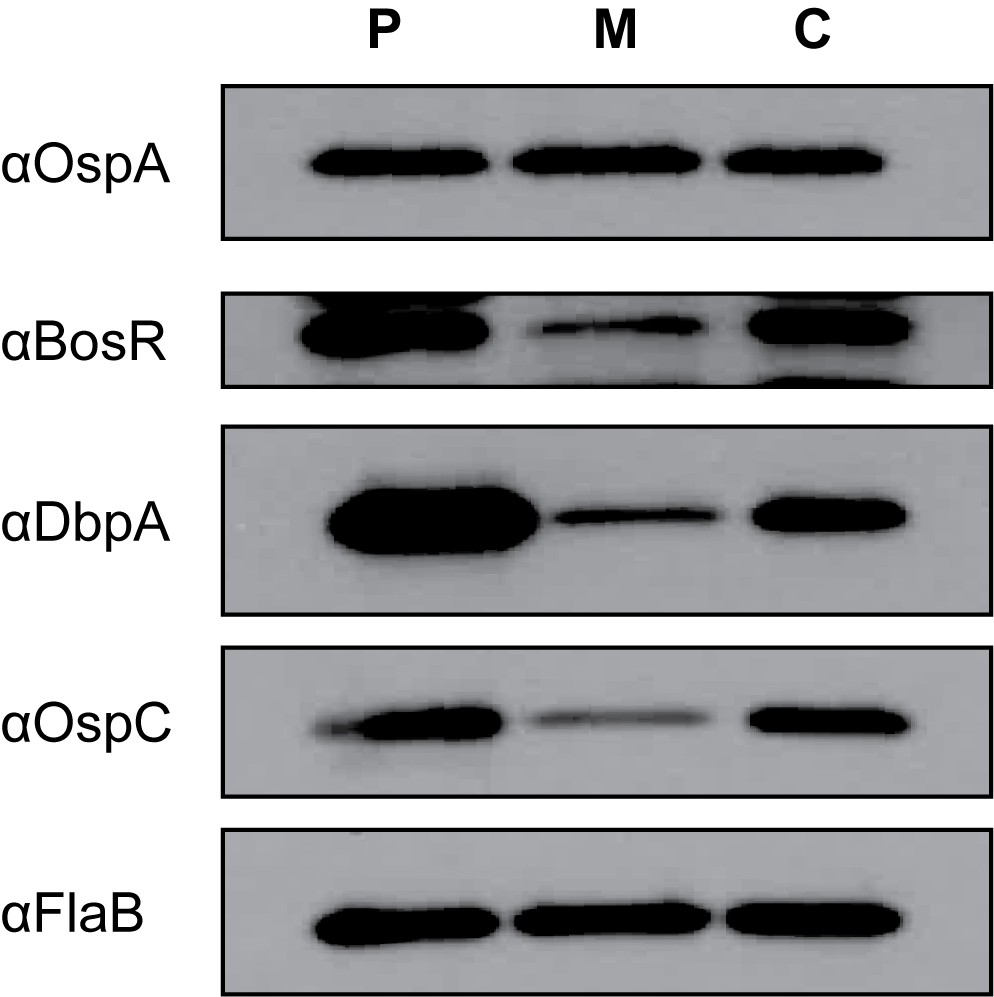

Supplement: Supplementary Figure 3 — Mammalian virulence factors production in bioluminescent cyaB mutant. B. burgdorferi ML23 strains were grown in BSK-glycerol to mid-log phase at 32°C 1% CO2. Protein was harvested and resolved on a SDS-PAGE with each lane containing approximately 4 × 10^7^ B. burgdorferi. Immunoblots were probed using the anti-serum depicted. FlaB was used as a loading control. Representative of at least three individual replicates. The following abbreviations are used to indicate strains: ML23 pBBE22luc (P), JH441 pBBE22luc (M), JH446 pBBE22luc (C). [file Image_3.TIF]
