# Supplementary material for: The Borrelia burgdorferi Adenylate Cyclase, CyaB, Is Important for Virulence Factor Production and Mammalian Infection
Source: Front Microbiol. 2021 May 25;12:676192. doi: 10.3389/fmicb.2021.676192 (PMC8186283; doi:10.3389/fmicb.2021.676192)
Supplement: Supplementary Table 1 — Primers used in this study. [file Table_1.docx]

**Supplemental Table 1.** Primers used in this study.

| **Primer Name** | **Sequence (5’ to 3’)** | **Purpose** |
| --- | --- | --- |
| *cyaB* US F | CAACTCAACTTTACAGAGTCTGTTC | Cloning |
| *cyaB* US R BamHI KpnI | ACGCGGATCCACGCGGTACCCTGAATTACTTTCATTGGCAAATCAAAG | Cloning |
| *cyaB* DS F KpnI SphI | ACGCGGTACCACGCGCATGCATATTAAAAATAATGTAATTATG | Cloning |
| *cyaB* DS F BamHI | ACGCGGATCCGTGAATGCCTAAATTACTAAGTC | Cloning |
| P*flgB*F-SphI | ACGCGGTACCCTAATACCCGAGCTTCAAGG | Cloning |
| *aadA*R-KpnI | GCGTGCATGCCAGATCCGGATATAGTTCCTCC | Cloning |
| *cyaB*FLAG-F-NotI | ACGCGCGGCCGCCCTTTGAAATAGAATCAAAAGC | Cloning |
| *cyaB*FLAG-R-XhoI | ACGCCTCGAGGTGCTGACATTGGGCTTTAT | Cloning |
| BbgenupF BamHI | GGATCCTATGCCTATGCAAAAAGCAG | Cloning |
| BbgenupR HpaI ClaI | GTTAACATCGATCAAAAAGCAGCTTGCAAATA | Cloning |
| BbgendownF HpaI ClaI | GTTAACATCGATTATGGCAGAGCTTGCATTAT | Cloning |
| BbgendownR KpnI | GGTACCGCAAGTGAAAACTCAAAACTTG | Cloning |
| pFlggentF-HpaIMCS | GTTAACGACGTCGACTGCAGTACTGAACGAATT | Cloning |
| PFlggentRHpaINotI | GTTAACGCGGCCCCGAGCTTCAAGGAAGA | Cloning |
| *bb0445*-F-BamHI | ACGCGGATCCATGTTTGGTTTTGATTTAATAA | Cloning |
| *bb0445*-R | ACGCGGATCCATGTTTGGTTTTGATTTAATAA | Cloning |
| P*flgB*-F-NotI | ACGCGCGGCCGCTAACGACGTCGACTGCAGTA TACCCGAGCTTCAAGGAAGATTTCCTATTAAG | Cloning |
| gent-R | CTGCTTTTTGTTAGGTGGCGGTACTTGGGT | Cloning |
| P*cyaB*-F-SalI | ACGCGTCGACATTAAACCTATCATTTCAATTG | Cloning |
| P*cyaB*-R | TGTAGTCCATATATTAAAAATAATGTAATTATGAT | Cloning |
| *cyaB*ORF-F | TTTTTAATATATGGACTACAAGGACCACGACGGCG | Cloning |
| *cyaB*ORF-R-SalI | ACGCGTCGACTTATTTTTTACTTTGATTTG | Cloning |
| *cyaB*SR0623-R-SalI | ACGCGTCGACGTGCTGACATTGGGCTTTAT | Cloning |
| pJH446-F PstI | ACGCCTGCAGCCCCAAGCTGGATTAGCAAC | Cloning |
| pJH446-R PstI | ACGCCTGCAGTGAGGACAATAATAATGTGAG | Cloning |
| P*flgB*-F-SalI | ACGCGTCGACTACCCGAGCTTCAAGGAAGA | Cloning |
| P*flgB*-R | GTAACATATAGAAACCTCCCTCATTTAAAAT | Cloning |
| GentR-F | GGGAGGTTTCTATATGTTACGCAGCAGCAAC | Cloning |
| GentR-R-AatII | ACGCGACGTCTTAGGTGGCGGTACTTGGGT | Cloning |
| *cyaB*FLAG-F-NdeI | ACGCCATATGATGGACTACAAGGACCACGA | Cloning |
| *cyaB*FLAG-R-HindIII | ACGCAAGCTTTTATTTTTTACTTTGATTTGCC | Cloning |
| *bb0722* RT F | GTAGCGATTCCCTGAAAGC | RT-PCR |
| *bb0722* RT R | CCTTCCATTTCAACATTAGGAC | RT-PCR |
| *bb0723* RT F | GTTTGAAATAGAATCAAAAGC | RT-PCR |
| *bb0723* RT R | CAGAGTAAGGTCTAGTTTC | RT-PCR |
| *bb0724* RT F | CCTGAAGCTATAGTTGTGG | RT-PCR |
| *bb0724* RT R | CCTTCCAATTGCCAGATCC | RT-PCR |
| *cyaB* F | AGACAACAACAATACTGTAGAAA | qRT-PCR |
| *cyaB* R | TTATTTCGTTTATCTCTACATTTA | qRT-PCR |
| *flaB* F | CAGCTAATGTTGCAAATCTTTTCTCT | qRT-PCR |
| *flaB* R | TTCCTGTTGAACACCCTCTTGA | qRT-PCR |
| *bosR* F | ACCCTATTCAACTTGACGATATTAAAGAT | qRT-PCR |
| *bosR* R | GCCCTGAGTAAATGATTTCAATAGATT | qRT-PCR |
| *dbpA* F | CAGATGCAGCTGAAGAGAATCCT | qRT-PCR |
| *dbpA* R | ACCCTTTGTAATTTTTCTCTCATTTTT | qRT-PCR |
| *badR* F | ACGCACTGCTGAACTTTCGATTTGGT | qRT-PCR |
| *badR* R | ACGCAGCATATTGACACAACCCTT | qRT-PCR |
| *plzA* F | ACGCGGATGTCGAGGAAGATGCAA | qRT-PCR |
| *plzA* R | ACGCAAAGCAATACCAAGCGCAAA | qRT-PCR |
| *rpoS* F | ACGCATGCAAACTTGCGACTTGTT | qRT-PCR |
| *rpoS* R | ACGCATCCCAAGTTGCCTTCTTGA | qRT-PCR |
| *ospC* F | CGGATTCTAATGCGGTTTTACTTG | qRT-PCR |
| *ospC* R | CAATAGCTTTAGCAGCAATTTCATCT | qRT-PCR |
| *rrp1* F | AAGGTGCTTACGAGATTGAG | qRT-PCR |
| *rrp1* R | TCTGTGGAACTTCTTGAACTAA | qRT-PCR |
| *hk1* F | CGTCAATTTATTTTCTAAGGATATTTTC | qRT-PCR |
| *hk1* R | TGCTTCGTCTTCAATTTCACT | qRT-PCR |
| *ospA* F | GCAACAGTAGACAAGCTTGAGC | qRT-PCR |
| *ospA* R | GTGTGGTTTGACCTAGATCGTCA | qRT-PCR |
| *bbk32* F | GAATATAAAGGGATGACTCAAGGAAGTT | qRT-PCR |
| *bbk32* R | TTTGGCCTTAAATCAGAATCTATAGTAAGA | qRT-PCR |
| *recAB* F | GTGGATCTATTGTATTAGATGAGGCT | qRT-PCR |
| *recAB* R | GCCAAAGTTCTGCAACATTAACACCT | qRT-PCR |
| Bactin F | ACGCAGAGGGAAATCGTGCGTGAC | qRT-PCR |
| Bactin R | ACGCGGGAGGAAGAGGATGCGGCAG | qRT-PCR |
| SR0623 5’ probe | GCCAATGAAAGTAATTCAGAGTAAGGTCTAGTTTCAATG | Northern |
| SR0623 3’ probe | GCCCTCAGATTGGAATTTATGGCAATCAAGGGCTTGTAATCTCTAC | Northern |
